# Recognition of Typical Locomotion Activities Based on the Sensor Data of a Smartphone in Pocket or Hand

**DOI:** 10.3390/s20226559

**Published:** 2020-11-17

**Authors:** Markus Ebner, Toni Fetzer, Markus Bullmann, Frank Deinzer, Marcin Grzegorzek

**Affiliations:** 1 Faculty of Computer Science and Business Information Systems, University of Applied Sciences Würzburg-Schweinfurt, 97070 Würzburg, Germany; toni.fetzer@fhws.de (T.F.); markus.bullmann@fhws.de (M.B.); frank.deinzer@fhws.de (F.D.); 2 Institute of Medical Informatics, University of Lübeck, 23562 Lübeck, Germany; grzegorzek@imi.uni-luebeck.de

**Keywords:** activity recognition, accelerometer, gyroscope, smartphone, sensor fusion, indoor localization, locomotion, principal component analysis, codebooks, support vector machine, nearest neighbor

## Abstract

With the ubiquity of smartphones, the interest in indoor localization as a research area grew. Methods based on radio data are predominant, but due to the susceptibility of these radio signals to a number of dynamic influences, good localization solutions usually rely on additional sources of information, which provide relative information about the current location. Part of this role is often taken by the field of activity recognition, e.g., by estimating whether a pedestrian is currently taking the stairs. This work presents different approaches for activity recognition, considering the four most basic locomotion activities used when moving around inside buildings: standing, walking, ascending stairs, and descending stairs, as well as an additional messing around class for rejections. As main contribution, we introduce a novel approach based on analytical transformations combined with artificially constructed sensor channels, and compare that to two approaches adapted from existing literature, one based on codebooks, the other using statistical features. Data is acquired using accelerometer and gyroscope only. In addition to the most widely adopted use-case of carrying the smartphone in the trouser pockets, we will equally consider the novel use-case of hand-carried smartphones. This is required as in an indoor localization scenario, the smartphone is often used to display a user interface of some navigation application and thus needs to be carried in hand. For evaluation the well known MobiAct dataset for the pocket-case as well as a novel dataset for the hand-case were used. The approach based on analytical transformations surpassed the other approaches resulting in accuracies of 98.0% for pocket-case and 81.8% for the hand-case trained on the combination of both datasets. With activity recognition in the supporting role of indoor localization, this accuracy is acceptable, but has room for further improvement.

## 1. Introduction

The ubiquity of smartphones has made many use-cases practicable, which were previously thought unfeasible. Thanks to their general versatility, the need for special equipment in many different areas of daily living almost completely disappeared. Due to their constantly increasing processing power, as well as the steadily growing repertoire of contained sensors and measuring devices, smartphones have also attracted the attention of many fields of research.

An example is the field of activity recognition, which tries to classify physical activities, such as walking or ascending stairs, currently performed by a person, based on sensor data. An extensive overview and introduction of the field of activity recognition using inertial sensors of either wearables or smartphones is provided in [1,2]. In earlier works, this was mostly done using multiple wearable sensors attached to several positions on the human body [3,4]. With the inception of smartphones, the focus of this field shifted to using them as single location sensor sources. For the employed approaches, this brought a whole new set of required properties, such as orientation- and location-independence, since people tend to carry their smartphones in locations of their preference. Possible use-cases for activity recognition range from logging activity diaries, to acting as information source for other applications, such as sports apps or other fields of research. Indoor localization is one such field that benefits from activity recognition as source of information. Research projects exist, attempting to implement indoor localization solely based on sources for relative position information, such as activity recognition and pedestrian dead reckoning, in combination with a building’s floor plan [5]. However, such techniques without sources for the absolute location within the building suffer from obvious disadvantages, such as the requirement to know a starting point or the inevitable drift over time.

Nowadays, indoor localization research often uses radio signals from existing Wi-Fi or Bluetooth infrastructure to estimate the absolute position of a person [6,7,8]. However, these signals are susceptible to numerous dynamic influences, such as people passing by, changing nearby interior, or opened and closed doors [9]. These influences cause strong fluctuations on the estimation, which is the main reason for why state of the art indoor localization systems are based on a combination of sources for absolute location information, such as radio signals, and sources of relative information, such as activity recognition and pedestrian dead reckoning [10,11,12,13,14]. In this combination, the source of relative localization information provides short-term accuracy, and the source for absolute localization information prevents the estimation from drifting off in the longer term. When activity recognition is used as part of this environment, several requirements should be fulfilled. First, the activity recognition should work in the most prominent positions, in which a smartphone is typically carried, for scenarios of indoor localization. For context-awareness applications, this mostly consists of passive positions, such as trouser pockets (called pocket-case in the remainder of this work), whereas navigational use-cases mostly consists of the smartphone being carried in hand, such that the display can be seen (hand-case). Second, the activity recognition should provide estimations with a latency as short as possible, since its main goal is to provide short-term accuracy. Third, since activity recognition is only one part of the overall application, it must not be overburdening resources, such as computational complexity, memory, and battery. In addition, activity recognition should be able to detect when the data it provides is not reliable. This, for example, includes situations where users start messing around with their mobile phones. In addition, finally, a practical implementation must be able to work with the hardware of smartphones that are most common today. This not only refers to the available computing power, but also to the selection of the available sensors.

The aim of this work is to find an approach for activity recognition that recognizes the typical locomotion activities in both pocket-case and hand-case in a as stable and accuracy way as possible. For that, we limited the focus on the four most basic locomotion activities used when moving around inside buildings: standing, walking, ascending stairs, and descending stairs, as well as the additional messing around class for rejections. Additionally, only accelerometer and gyroscope will be used, since the barometer has great potential, but only a small distribution among the currently distributed smartphones. To find a suitable approach for our use-case, we will introduce a novel approach based on analytical transformations combined with artificially constructed sensor channels, and compare that to two approaches adapted from existing literature, one based on codebooks, the other using statistical features. The latter was also included to serve the purpose of comparability with other works, as it maps best to existing research for the pocket-case.

The paper is structured as follows: In Section 2 we examine papers with a close relation to our work. Next, Section 3 contains an introduction of the employed sensors and coordinate systems, as well as short discussions on the activities to be identified, together with their characteristics and possible difficulties in both cases. The used activity recognition approaches are presented in Section 4, followed by evaluation and comparison between them in Section 5. Finally, the work concludes in Section 6.

## 2. Related Work

Activity recognition has been a field of research for many years already. Most works in this area differ in their set of selected activities, while having the basic locomotion activities, which are covered in this work, as common basis most of the time.

One of the first works on the general subject of activity recognition is [15]. Like most research before the inception of smartphones, it focuses on activity detection using wearable sensors. For the activity recognition using wearables, the publications of Bulling et al., as well as the publication by Lara and Labrador, are the established works of reference [2,16]. Most of this research is based on the use of multiple sensors placed at different positions on the human body, which is rather impractical for real-world scenarios. Though, mostly for the detection of complex activities such as opening a door, wearable sensors are still being researched today [17].

The publications of Shirahama et al., using a codebook approach together with six accelerometers rank among the best with regards to accuracy [18,19] in this area. Over the course of this work, the codebook approach will be adapted to and tested for the smartphone use-case at hand. Due to the lack of practical uses with many wearables, Lester et al. investigated whether this large number of wearables, which were common in most publications before, are really necessary for the detection of the most common activities of daily living [20]. As a result of the reduction in the number of sensors, the topics of location- and orientation-independence arose, to allow a user to pick their favorite position for the sensor. Though, the increasing popularity of smartwatches makes activity recognition with a single wearable an interesting target for research [21]. These properties are also essential for activity recognition using smartphones, since users typically only have a single smartphone, which they carry in a position of their personal preference. With the inception of smartphones, the number of possible use-cases for activity recognition increased [22]. Most of the studies in this area deal with the case of smartphones carried in passive positions such as pockets or even handbags. Good general overviews of activity recognition with smartphones can be found in [22], which mainly focuses on challenges and possible use-cases, as well as [23,24], which give a general technical overview of approaches and other works in this area. The hard constraint of only one sensor location, as well as the necessity for orientation-independence is the subject of multiple works, such as [25,26]. The topic of user independence is further researched in [26,27]. Commonly represented in publications focused on smartphones is the use of the gyroscope sensor. Ref. [28,29] examine the roles of the different sensors for activity recognition, also examining relations to single activities. They conclude that the magnetometer leads to barely any improvements when used in addition to accelerometer and gyroscope.

Most smartphone-based publications use approaches based on hand-crafted statistical features, instead of feature learning approaches [26,30,31,32,33], while also doing a comparison between classification approaches such as SVM, Naive Bayes, and decision trees. Ref. [34] gives an overview of many commonly used features applicable to accelerometer data. This approach will also be discussed in this paper, while using a handpicked combination of features from the ones used in the mentioned works. As briefly mentioned earlier, the most works in activity recognition with smartphones cover the case of devices carried in passive locations, while the case of a smartphone being statically held in hand as would be the case for pedestrian navigation use-cases has very little coverage in activity recognition research yet. Ref. [5] is one of the few publications with a similar use-case, which is directly using the recognition of activity sequences for localization, though while making use of a barometer. Ref. [35] is another publication that uses data from a smartphone carried in the hand. However, the work leaves open an exact description of the data acquisition, and from the existing description it can be assumed that the smartphone was merely held in the hand while performing the usual locomotion arm movements.

## 3. Sensors and Locomotion Activities

### 3.1. Sensors

**Figure 1:**
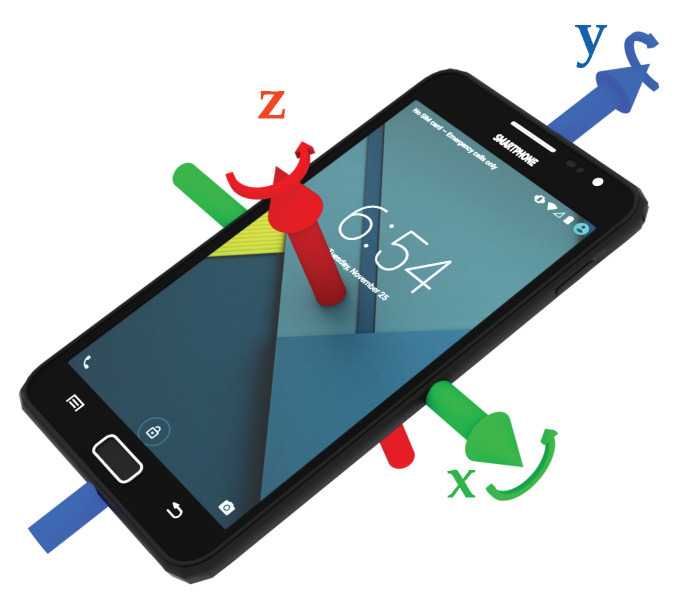
Coordinate system used for sensor data measured on smartphones using the iOS or Android operating systems.

For activity recognition, multiple sensors of a smartphone can provide valuable information. Previous studies, as mentioned in Section 2, have successfully used accelerometers, gyroscopes, and barometers. Due to the low availability of barometers, this work focuses on the combination of accelerometer and gyroscope. Most sensors in Android and iOS use the same coordinate system, as shown in Figure 1. This coordinate system is typically called device coordinate system. Accelerometers measure the acceleration along the three depicted axes, mostly consisting of a static and a dynamic component:

(1) $$a={\left(a_{x},a_{y},a_{z}\right)}^{⊺}=a_{\mathrm{static}}+a_{\mathrm{dynamic}}+a_{\mathrm{noise}}\;\mathrm{where}\;a_{\mathrm{static}}=O_{E\to L}\;\cdot \;-g,$$

where $a_{\mathrm{static}}$ is equivalent to the negative gravitational acceleration, rotated from the earth’s coordinate system into the device’s local coordinate system by $O_{E\to L}$, $a_{\mathrm{dynamic}}$ is the acceleration applied on the phone by the user, and the sensor’s noise $a_{\mathrm{noise}}$. This, however, also means that the device coordinate system depends on how the smartphone is currently oriented. If the user changes the device’s orientation, collected acceleration data also is rotated. Orientation sensitivity is an undesirable property in activity detection, since there is no correlation between a device’s general orientation and the currently executed activity. This is why many publications deal with this topic, in one form or the other [23]. Aligned coordinate systems can help to remedy this problem, by deriving a deterministically calculatable transformation from the sensor data in the device coordinate system to a coordinate system independent of the smartphone’s orientation. Since the transformation between device- and aligned coordinate system has to be derivable from the raw sensor data, most of the aligned coordinate systems try to align one of the axes to earth’s gravity vector, which is a good absolute measure of the device’s orientation. The most basic approach to calculate this orientation is to isolate the gravity vector from the measured acceleration with a long-term mean, and then determining the rotation $O_{L\to E}$ from the isolated gravity vector in the device coordinate system, to gravitation in the earth coordinate system ${\left(0,1,0\right)}^{⊺}$. However, since this introduces a large delay, practical use-cases typically use more advanced techniques such as complementary filters [36,37], or the Madgwick filter [38,39], which directly calculate $O_{E\to L}$ using a combination of accelerometer for long-term stability, and an integration of the gyroscope for quick orientation changes. Since both approaches use only one reference for the alignment, the remaining two coordinate system axes remain unspecified, and are allowed to rotate by sensor inaccuracy. Acceleration $\hat{a}$ in this underspecified earth coordinate system is calculated using:

(2) $$\hat{a}=O_{L\to E}\;\cdot \;a\;\mathrm{where}\;O_{L\to E}={O_{E\to L}}^{-1}$$

Aligning the two mentioned remaining axes to another fixed reference in the earth coordinate system, such as one of the magnetic poles, is more of a hindrance than helping, since that would introduce a sensitivity to the walking direction. To fix this, Yang has instead chosen to represent measured accelerometer data as two-dimensional [25,40], consisting of a vertical and a horizontal component. Movement along the horizontal axis is then only represented by its magnitude, intentionally discarding orientation information. Acceleration $a^{\prime }$ in this two-dimensional coordinate system is calculated using:

(3) $$a^{\prime }={\left({\hat{a}}_{y},\sqrt{{\hat{a}}_{x}^{2}+{\hat{a}}_{z}^{2}}\right)}^{⊺}.$$

The gyroscope, as second used sensor in this work, measures the current angular velocity around the shown axes in rad/s, which is composed of the sensor’s noise $\theta_{\mathrm{noise}}$ and the angular velocity caused from movements of the user $\theta_{\mathrm{dynamic}}$. A gyroscope measurement is given by:

(4) $$\theta ={\left(\theta_{x},\theta_{y},\theta_{z}\right)}^{⊺}=\theta_{\mathrm{dynamic}}+\theta_{\mathrm{noise}}.$$

As both Equations (1) and (4) show, accelerometer and gyroscope each have a noise component in the measured data. Part of this noise is normally distributed noise, which is less significant for the gyroscope, when compared to the noise measured by typical MEMS accelerometers. This makes gyroscopes very reliable as short-term component in combinatory techniques such as the complementary filter. Another significant part of the noise is a bias, which is caused by properties of the underlying MEMS-technology of both sensors [41]. Android and iOS try to remove this bias internally, using techniques such as the ones discussed in [42], which has proven to be sufficient for the requirements in this publication.

### 3.2. Locomotion Activities

**Figure 2:**
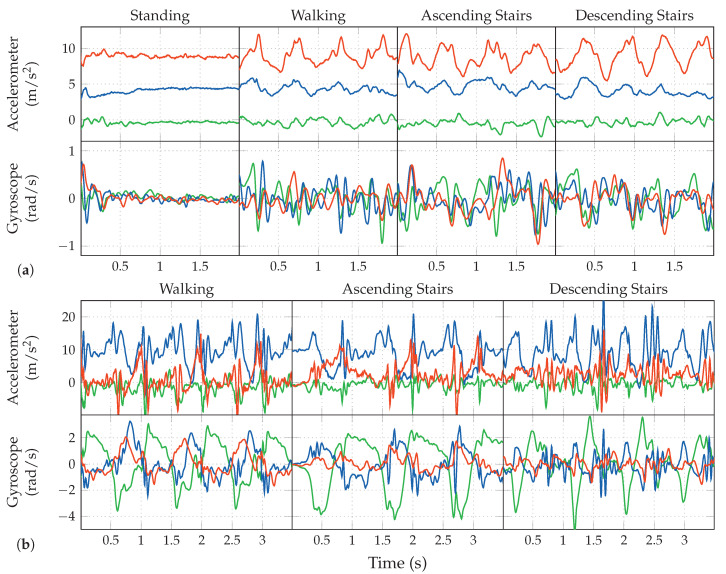
Raw sensor data sequences of the covered activities in hand-case (**a**) and pocket-case (**b**). Green, blue, and red represent the device’s local coordinate system axes x, y, and z respectively.

Based on the example data shown in Figure 2a,b, we will next do a short discussion of the activities to be detected for our use-case, their properties, characteristics, as well as a comparison between pocket-case and hand-case.

*Standing*. As one would expect, the standing activity does not exhibit any periodic behavior within the measured data. This is in contrast to the other covered activities, which is also clearly visible in the shown samples of Figure 2a,b. In the possible use-case of doing a navigation within a museum, standing could additionally comprise the act of looking around. Most of the times, looking around does not exhibit longer sequences of periodic behavior either, but a simple check for any action in the measurements to decide whether the current activity is standing, might fall into the trap of rejecting this situation. In the case of simply no action in the signal, there is barely any difference between the hand-case and the pocket-case. The standing activity has thus not been included again in Figure 2b. Simply looking around mostly involves one’s upper body, which results in little movement to be recorded in the lower body, to which the pocket is indirectly attached. The problem of looking around is thus less interesting for the pocket-case.

*Walking*. In the measurements, any activity that involves walking can be recognized by its characteristic periodic behavior. The exhibited frequencies caused by gait are dependent on many factors; one of them being the smartphone’s location. When the phone is held in hand, this periodic behavior is mainly visible in the accelerometer measurements, whereas the most characteristic part of the signal is found in the gyroscope measurements, when the phone is carried in a trouser pocket. Additionally, the exhibited frequency for the hand-case appears to be roughly double that of the trouser pockets case. This is because the most evident part of the periodic behavior in the hand-case is the up- and downwards movement of the upper body, caused by the steps. Here, the smartphone is able to measure each step, while when attached to the trouser-pocket, it mostly measures the movement of only that single leg (mainly in the gyroscope’s x-axis in Figure 2a). Due to this difference, Figure 2b visualizes data over a longer time span than Figure 2a. Additionally, people walk at different speeds, which introduces an unknown in the exhibited frequencies [43], further obscured by factors such as a person’s mood, situation, and age.

**Figure 3:**
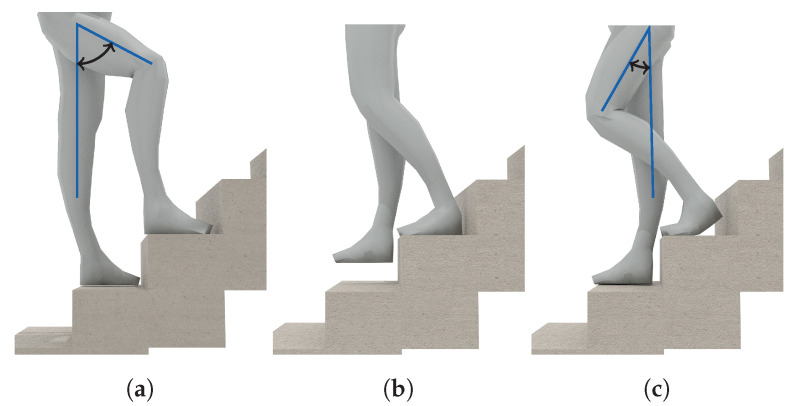
Typical poses and movements exhibited while walking stairs. (**a**) demonstrates the high inclination typically exhibited during the ascend of stairs. (**b**,**c**) demonstrate the typically much smaller thigh inclinations during the descend of stairs.

*Walking Stairs*. During normal gait, the maximum inclination of the thighs is rather small, because they are the heaviest part of the leg, and raising them costs energy. When climbing stairs, this maximal inclination is higher than for normal gait. As demonstrated in Figure 3a, the angle of inclination is mainly important for the pocket-case. Since the gyroscope only measures the angular velocity, and not the actual angle, comparing the walking activity with the activity of ascending stairs in the samples at Figure 2b is not trivial. The most significant difference between both is the length of their peaks exhibited on the gyroscope’s x-axis, which, however, also depend on step frequency. In the hand-case, the differentiation between walking and ascending stairs from the samples shown in Figure 2a seems to be hard, since there is no apparent constant visible difference, apart from the frequency that is slightly faster when walking on an even surface. However, as mentioned above, making this differentiation by means of calculating the exhibited frequency is bound to be unstable even on a per person basis.

For the activity of descending stairs in the hand-case, the differentiation bears pretty much the same problems. In the pocket-case, the differentiation seems easier. As shown in Figure 3b,c, the maximum inclination of the thighs while descending stairs is much smaller than during ascend, because most of the rotation is absorbed by bending the toes on the previous stair step. Thus, when only looking at the observed maximal inclination of the thighs, this makes descending stairs very similar to normal gait. However, as seen in Figure 2b, the shape of the angular velocity has sharper and shorter peaks, which are probably caused by the smaller step size compared to normal gait, as well as the support of gravitation during the downwards movement.

*Messing Around*. If indoor localization on the smartphone is not used for the mere guidance to a destination, another aspect appears that needs to be considered. Sometimes, users start to mess around with their smartphones during idle periods, which could for example involve hitting the smartphone against their thighs to the beat of the music in a department store while standing in front of a shelf. There is no fixed pattern for the way users play around with their smartphones that could be easily trained. Handling this kind of activity would need to be done by detecting outliers from the normally occurring values, a rejection class, or similar mechanisms. Since the detection of activities has to be orientation independent, deciding if a user is messing around by focusing on certain axes of sensor data is not a reasonable solution.

## 4. Activity Recognition

Activity Recognition is the task of classifying a continuous stream of data. For this reason, only a temporally limited segment of the continuous signal at the input is considered at any time. This is achieved by using the Sliding Window approach on the sensor data streams in each of the three approaches examined throughout this work. One instance of data within such a temporal window at one point in time will be called sequence in the rest of this work. The longer these temporal windows, the worse the classification of very short activity sequences, such as stairs consisting of only three steps, will be. Whereas a temporal window that is too short will at some point have the problem of not containing enough information for a reliable classification. This length thus is a tradeoff between temporal accuracy and classification accuracy. It further also depends on the user, since older people are generally moving slower and therefore need a larger window than younger users, to cover periodic movements. Based on the findings of previous studies [44,45] this work is using sequence lengths between 2 s and 3 s for all approaches.

### 4.1. Analytical Transformations

Based on analytical transformations, this novel approach first tries to learn a linear transformation suitable for dimensionality reduction on raw, as well as computationally produced additional sensor data. Using an analytical transformation for dimensionality reduction here has the potential to provide better class separability, while also reducing the computing time required in the classifier. Since applying the rotation matrices calculated by the transformation on the data is comparatively cheap, this results in a reduction of the overall approach’s computational complexity. As analytical transformations, we tested both a PCA [46] and an LDA, which is also often called Fisher Discriminant Analysis [47].

**Figure 4:**
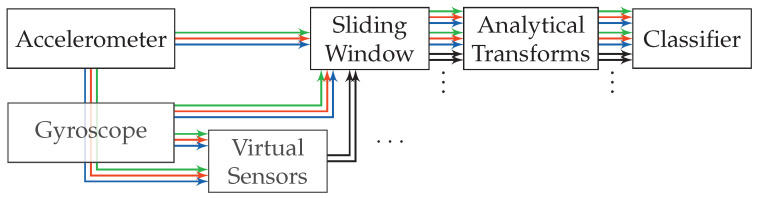
Flow diagram of the pattern recognition pipeline employed for the approach based on analytical transformations. Accelerometer and gyroscope each output the three streams x-axis (green), y-axis (blue), z-axis (red), which are processed independently throughout the pipeline until their features are concatenated for classification. Virtual sensors supply additional, artificially created, sensor channels (black). No preprocessing is required for this approach.

The approach’s pattern recognition pipeline is shown in Figure 4. It uses the raw incoming sensor data of gyroscope and accelerometer, of which all sensor channels (x-, y-, and z-axis) are handled independently. On each of the sensor channels, a sliding window is used to extract sequences. All of these separate sliding windows are synchronized across sensor channels. All associated windows extracted at one point in time on the stream will in the following be summarized under the term *time-window*. One analytical transformation is used per sensor channel, resulting in six unique transformations when gyroscope and accelerometer provide three channels each. Each of these analytical transformations is used on the raw sequences of sensor data, which were extracted using the sliding window on each sensor channel. These transformations significantly reduce the dimensionality of the sequences, extracted from their respective sensor channels, and at the same time provide the potential of improving separability.

To train the transformations, all activity sequences from the recordings are run through the pipeline up until the analytical transformations. For this, the sequences extracted from a labeled dataset are separately accumulated per sensor channel, further broken down by the contained activity class Ω. These |Ω| activity sets, accumulated per sensor channel, are then used to train the respective analytical transformation for each sensor channel. When the sensor data is sampled with 50Hz, a sliding window of length 2 s thus results in 100 samples per extracted sequence, which is seen as 100-dimensional vector of the corresponding analytical transformation. Smartphones are limited in their calculation, as well as their battery capacity, even more so since activity recognition is only one task in the background for most use-cases. The assumption is that the sequences contain a significant amount of correlation, which can be reduced by an analytical transformation without loosing too much of the contained information. After this dimensionality reduction has been done per sensor channel, all resulting feature vectors will be concatenated to one big feature vector per time-window, which is then used for classification. For an exemplary reduction to 3 remaining dimensions per sensor channel using accelerometer and gyroscope, this would result in a concatenated feature vector of length 6×3=18, when two sensors, and thus six sensor channels are used as input.

Conceptually, calculating the PCA tries to learn template sequences (eigenvectors), which can then be used to describe new encountered sequences during operation through a linear combination of the templates learned from the training dataset. The result of this linear combination to reconstruct a newly encountered sequence from the live stream during operation is a histogram-like feature vector describing the prevalence of each of the learned template sequences in the linear combination. This makes noise filters unnecessary, since the resulting eigenvectors are the ones with the largest variance, and thus only contain a miniscule amount of white noise. The sensor bias, which is mainly a problem for the accelerometer, turned out to be unproblematic. Most of the bias is corrected by the online calibration done in Android.

**Figure 5:**
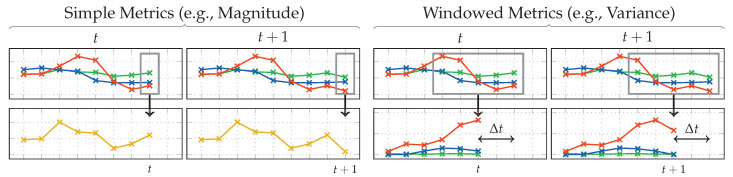
Calculation of virtual sensors for simple and windowed metrics at the example of magnitude and variance, simulated for two consecutive time-steps. For a window size of $w_{\mathrm{virt}}$, windowed metrics have a delay of $\Delta t=\frac{w_{\mathrm{virt}}-1}{2}$, while simple metrics such as magnitude always have a delay of Δt=0.

Publications such as [48] from Khan et al. suggest that the problem cannot be easily solved by linear approaches. They use a KDA for dimensionality reduction and for improving separability. This is a variation of the LDA used in this work, extended by applying the kernel trick [49]. In case of analytical transformations, however, the kernel trick makes their memory complexity often scale quadratically with the number of samples used for training in practice, instead of the number of dimensions. For big, representative datasets, this is impractical. Instead, in an attempt on getting this approach to gain non-linear separation abilities, the concept of virtual sensors was adopted. These virtual sensors generate additional streams of data by taking raw sensor data as input, and continuously calculating metrics, such as the magnitude, on them. Using $\sqrt{{a[t]}^{⊺}a\left[t\right]}$ for each raw accelerometer sample a[t] at time *t*, simple metrics such as the magnitude can be calculated on a per-sample basis without delay. More complex metrics, such as variance on the other hand, always require multiple samples as input. For this, a sliding window with a length of $w_{\mathrm{virt}}$ is used that moves across the raw stream of sensor data using a step width of 1. In pre-tests, $w_{\mathrm{virt}}\approx 0.3\;s$ has shown to perform best while allowing a small delay. This happens before a sliding window approach is used to extract temporal sequences for each time-window, and is thus not to be confused with those later in the pipeline. Due to this window, these windowed metrics introduce a delay of $\Delta t=\frac{w_{\mathrm{virt}}-1}{2}$. The chosen metrics further also differ in the amount of input and output dimensions. Calculation of simple and windowed metrics at the examples of magnitude and variance is demonstrated in Figure 5. An overview of all used virtual sensors and their metrics is given by Table 1. For the remaining pipeline, including the extraction of sequences, virtual sensors look and behave no different than real sensors.

The calculated virtual sensors and their corresponding metrics were chosen to be beneficial for activity recognition. For example, magnitude has been used to achieve rotational invariance in works such as [28,29]. Step detection based on magnitude is much easier if the orientation of the smartphone is unknown, since the magnitude is independent of the orientation. Other metrics such as standard deviation, variance, and RMS, on the other hand, allow an easier differentiation between resting activities and activities which involve movement [30,34]. The Inclination metric listed in the table is a metric that calculates the absolute rotation of the device in its local coordinate system along x-axis (called TD, for Top-Down) and y-axis (called LR, for Left-Right) in rad. This metric is calculated on the accelerometer data on a per-sample basis using:

(5) $$f_{\mathrm{TD}}^{\left[t\right]}=atan2\left(a_{y}^{\left[t\right]},a_{z}^{\left[t\right]}\right)\;,\;\mathrm{and}\;f_{\mathrm{LR}}^{\left[t\right]}=atan2\left(a_{x}^{\left[t\right]},a_{z}^{\left[t\right]}\right),$$

where $f_{\mathrm{TD}}^{\left[t\right]}$ and $f_{\mathrm{LR}}^{\left[t\right]}$ denote the feature calculated at time point *t* along x-axis and y-axis respectively. The function atan2 is an extension of $\tan^{-1}$, which considers the quadrant in the calculation. One sample at time point *t* of the accelerometer data from the stream used for the calculation is denoted by $a_{x}^{\left[t\right]}$, $a_{y}^{\left[t\right]}$, and $a_{z}^{\left[t\right]}$ for the device’s local x-axis, y-axis, and z-axis respectively. After the virtual sensors have been calculated, and all streams of channels have been synchronized, the extraction of windows from each sensor-channel to produce features for a time-window is then done analogous to what has been described for the real sensors above. At that point in the pipeline, virtual sensor channels and real sensor channels are treated equally.

#### Outlier Detection

As discussed previously, messing around with the smartphone consists of very individual movements. Creating a dataset for messing around is thus not an option. It follows that the analytical transformations used in this approach cannot be trained using recordings of users messing around with their smartphone. Due to the manifold nature of movements done while messing around with a smartphone, there will certainly be a set of movements that are too similar to the actually covered activities in this work, thus leading to a certain amount of misclassifications. The approach described in this section uses the concatenated feature vectors calculated per time-window for outlier classification. Since no representative set of training data for outliers can be produced, a normal classification approach is impossible. Thus, a one-class classifier is used to classify whether a feature vector is an outlier or contains an actual activity sequence. For this task, a one-class Support Vector Machine (SVM) using the well-known Radial Basis Function (RBF) kernel is used. Finding a value for the hyperparameter γ of the kernel is done as part of the evaluation.

### 4.2. Codebook

One of the best performing approaches regarding accuracy in the field of activity recognition is the codebook approach by [19]. However, to the best of our knowledge, this approach has yet only been used in combination with wearable devices. The original work, which will be used as model for this work, used six accelerometer-only wearables mounted on different body positions. Conceptually, this approach tries to learn characteristic shapes exhibited in the sensor data during training. When the codebooks are then used during operation, exhibited shapes from the stream are compared against the learned characteristic shapes, which produces a histogram-like feature vector. Due to the clustering done in the training phase, no filtering is required on the raw sensor data. The following will describe our differences to the original, while adopting the terminology of sequences, subsequences, codebooks, and codewords from the mentioned publication.

**Figure 6:**
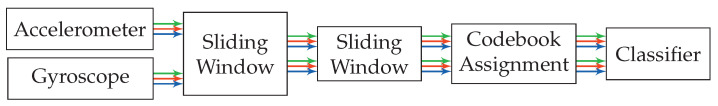
Flow diagram of the pattern recognition pipeline employed during operation of the codebook-based approach. The two sliding windows correspond to the ones employed by the codebook approach for the extraction of sequences and subsequences respectively. Accelerometer and gyroscope each output the three streams x-axis (green), y-axis (blue), z-axis (red), which are processed independently throughout the pipeline until one of the two codebook assignment strategies is used.

**Figure 7:**
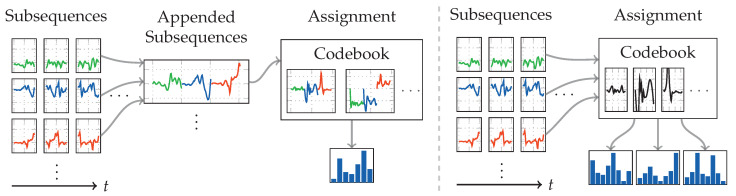
Diagrams visualizing the strategies for using a single codebook with multiple sensor channels as used by [19] (**left**) and us (**right**). With the left variant, subsequences from all channels are appended to a vector that is then used for codebook assignment, producing only one histogram feature vector per time-window and sensor. The variant on the right treats subsequences of all sensor channels equally, and assigns them using the same codebook, resulting in 3 independent histogram feature vectors.

Figure 6 shows a flow diagram of the recognition pipeline employed with the codebook approach. As can be seen, the first steps are sliding windows to extract sequences of length $w_{\mathrm{seq}}$, which are then further subdivided into subsequences of length $w_{\mathrm{sub}}$. Instead of multiple accelerometers at different locations, we use accelerometer and gyroscope as two different sensors, though within the same location. To capture correlation between sensor channels, the authors use a strategy that appends all temporally related subsequences of all sensor channels, before assigning this composite with the codebook. This workflow is visualized on the left side in Figure 7. We instead use the strategy depicted on the right, which does not append subsequences, and instead assigns them one by one using the same codebook. In a pre-test, this strategy produced slightly better results. The biggest difference between both strategies of combined sensor channels is the number of feature vectors produced per sensor. While the strategy without appending subsequences produces the same number of feature vectors as the sensor has channels, the appending strategy always only produces one feature vector.

Multiple variants for the assignment of sequences to a codebook exist. As [19] suggest, we use Soft Assignment, which has shown better general versatility. Additionally, the codebook approach has a couple of hyperparameters. These are the width of the extracted sequences $w_{\mathrm{seq}}$ with the corresponding step size, the width of extracted subsequences $w_{\mathrm{sub}}$ with the corresponding step size, as well as the codebook size |C|. For the Soft Assignment strategy, there additionally is σ, which controls the Kernel Density Estimation’s smoothness. The parameters |C| and σ have to be chosen with respect to use-case and dataset, which will be done as part of the evaluation. To reduce computational complexity, we used a fixed step width of $s_{i}=2$ samples for subsequences ω, which was determined empirically. The outer sequences s, which are divided into subsequences during operation, are not moved freely, but instead moved by a multiple of this subsequence step width. The length $w_{\mathrm{seq}}$ of sequences s is thus not given in samples directly, but instead implicitly determined through the number of subsequences $n_{\mathrm{sub}}$ contained. The step width used for sequences s is then given by $\frac{1}{4}n_{\mathrm{sub}}$, which moves sequences $\frac{1}{4}$th of their contained subsequences forward per time step. This alignment of sequences to the contained subsequences can in production be used to reduce computational complexity, since codebook assignments have to be done only once per subsequence, which can then be reused for 4 consequent sequences. Many approaches in the context of activity recognition use a step width in the same order of magnitude [3,27,32,50,51]. The smaller this step width $s_{o}$ for sequences, the smaller the delay of the approach, but the higher the computational complexity. The actual sequence length $w_{\mathrm{seq}}$ can then be calculated using:

(6) $$w_{\mathrm{seq}}=\left(n_{\mathrm{sub}}-1\right)s_{i}+w_{\mathrm{sub}}$$

Since the codebook approach is essentially based on learning characteristic shapes and trying to find them in new data, this approach has a good potential with regards to the pocket-case. As discussed in Section 3.2, the pocket-case exhibits a range of characteristic shapes for all activities in the raw sensor data. When looking at the results of the first study on activities’ exhibited shapes, done in the mentioned section, the hand-case might turn out to be a bigger problem for the codebook approach. While the differentiation between activities that involve walking (walking, stairs up, stairs down), and the standing activities based on shapes seems feasible; the differentiation between the walking activities based on shape seems difficult.

#### Outlier Detection

For outlier detection, the features calculated using the codebooks will be used. This could, however, prove difficult, since the codebook’s task is to find characteristic shapes in the trained signals to then try and differentiate between the activities. Though, since messing around does not have a dataset that could be trained, the deployed codebooks have no reference shapes to map the seen subsequences to, which are characteristic for messing around. During codebook assignment, the messing around subsequences will thus be assigned to the shapes in the codebooks that have been trained with the positive dataset. For the codebook, this problem is thus even more pronounced than for the one mentioned for analytical transformations in Section 4.1, since the codebook is limited to the codewords it has learned, while the approach based on analytical transformations does a linear combination with its learned sequences. And due to the employed normalization of the resulting histogram feature vector to a sum of 1, a differentiation between outliers and actual activity samples will have to be solely based on the probability distribution within the feature vectors. Main difference between some of the movements done while messing around with the smartphone and normal activities is their larger magnitude. Since the codebook is mainly comparing shapes, and invariant to the exhibited magnitudes in the shapes to a certain extend, these types of messing around could prove to be complicated to detect. As with the approach based on analytical transformations, a one-class SVM using the RBF kernel will be facilitated for this differentiation.

### 4.3. Statistical Features

**Figure 8:**
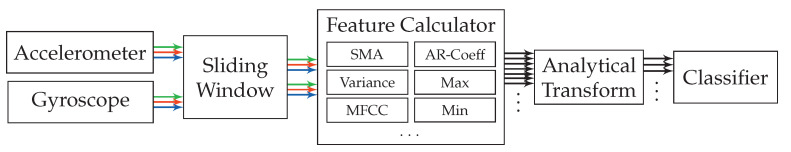
Flow diagram of the pattern recognition pipeline employed for the approach based on statistical features. Accelerometer and gyroscope output the three streams x-axis (green), y-axis (blue), z-axis (red), which are processed independently throughout the pipeline until their features are concatenated for classification.

The last examined approach for activity recognition is the most commonly used approach in activity detection and fall detection research [25,29,40,52]. As the comparison of numbers between papers is difficult due to different sets of activities to be identified, this approach can also be seen as representative for the comparison with other papers. Its basic principle is to calculate a set of features on all sequences that were extracted across sensor channels at one point in time using the sliding window. These calculated features are appended, and thus form a large feature vector for one point in time, on which classification can run. Figure 8 shows a flow diagram of the employed pattern recognition pipeline for this approach.

**Figure 9:**
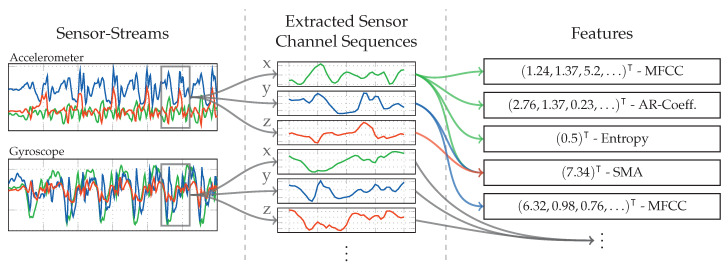
Flow diagram of the feature extraction workflow during operation of the approach based on statistical features using exemplary data and a subset of features. Sequences are extracted from each sensor channel. Features are calculated on the extracted sequences.

As with the other approaches, the set of sequences extracted at one point in time from the sensor channels, is summarized under the term *time-window*. Figure 9 visualizes the feature extraction workflow, where one feature vector is generated for each such time-window, by calculating a set of statistical features on the extracted sequences. Not all employed statistical features are calculated for the sequences of all sensors. Table 2 gives an overview of the mapping from calculated features to sensor channels, as well as the resulting amount of dimensions. Directly using the large vector of appended features for classification is impractical on a mobile device, due to its large amount of dimensions and the resulting computational complexity required. Therefore, one analytical transformation after the feature extraction is used to reduce the dimensions of this concatenated feature vector, before using it for classification. To train this analytical transformation, the pipeline is run with the activity sequences from the recordings as input. To also support class-aware analytical transformations, this is done separately for each of the activities, which results in one set of feature vectors per class Ω, on which the analytical transformation can then be computed.

As can be seen in the features listed in Table 2, some are calculated on sensor channels such as Accelerometer Magnitude, or Inclination Gradient, which do not actually exist in hardware. These streams are computed on the fly while calculating the actual feature on them, very similar to the virtual sensors concept. Thus, Magnitude and Inclination were adopted as presented in Section 4.1 for the approach based on analytical transformations. Additionally to these, the gradient sensor, which calculates the difference between two consecutive samples per channel of the incoming signal, was added. The selected features presented in Table 2 are a collection of the best performing ones, gathered from a large set of research on activity recognition. A good overview of commonly used features for accelerometer signals can be found in [34], while [23,24] provide a general overview of features used in a large set of publications for multiple sensors. The following will give a more in-depth view of the features we chose, as well as implementation details where appropriate.

*SMA and Variance* have shown to be valuable features for the differentiation between idling activities (low variance) such as standing, and active activities (high variance) such as walking. These are one of the most common features, also mentioned in [30,32,34,50,51,52,53].

*MFCC* is a set of features calculated in the frequency domain, widely used for audio fingerprinting [54]. The use of features in frequency domain has long been avoided by research on activity recognition, since its calculation was very costly and impractical on old smartphones. Instead, some publications used rough approximations of the examined base frequencies by counting peaks [55] or calculating the times between peaks [52,53]. On more modern smartphones, features in time domain are less problematic, which is why they will be used in this work in form of the MFCC features. Using a multi-dimensional feature in frequency-domain could prove helpful for the detection of non-periodic behavior in the sensor data. Since, while periodic movement with a consistent frequency produces a clean frequency spectrum with few and very significant peaks, un-periodic behavior tends to produce chaotic frequency spectra without peaks. MFCC feature calculation is configured using the two hyperparameters $n_{\mathrm{mel}}$ and ψ, controlling amount and growth of the filters in the used filter-bank respectively. Determining appropriate values for these hyperparameters on the given datasets is done as part of the evaluation.

*AR-Coefficients* are especially crafted for the prediction of repeating patterns, using them for activity recognition thus seems logical. These features have already been used in the context of activity recognition by publications such as [48]. The amount of calculated coefficients is controlled by the hyperparameter |q|, for which finding a suitable value is done in the evaluation. When used together with MFCC features, the autoregression coefficients could turn out to be redundant. The evaluation will thus do a test of different values for |q|, as well as a comparison against MFCC features.

*Entropy* provides an assessment on the activity contained in a signal, similar to SMA and Variance. The entropy uses a discrete histogram calculated on the exhibited values in each time-window. In this work, the underlying histogram has the size of 256 bins, which was empirically determined, since no implementation details were found in other publications that make use of the entropy [3,25,30,53].

*Correlation* delivers an indication of similarities in patterns found across multiple sensor channels. When Correlation is listed for one sensor source, it is calculated between all unique permutations of a sensors’ *n* channels, thus resulting in $\frac{n!}{2(n2)!}$ feature dimensions. This feature has been used by publications such as [3,25,30,32,50,51,53].

*75th Percentile, Min, and Max* are extreme values hinting at the strength of the recorded movement. This could be helpful for the outlier detection, since messing around might contain higher acceleration peaks for certain movements. These features have been used in [25,55]. The 75th Percentile in this work is calculated using linear interpolation on the observations in a time window.

#### Outlier Detection

As with the other two approaches, outlier classification is done using a one-class SVM with the RBF kernel trained and operated on the features calculated as part of the approach’s pattern recognition pipeline. The use of an analytical transformation for dimensionality reduction, however, could prove to be a problem. When the analytical transformation is trained on raw sensor channel data, as is the case for the approach based on analytical transformations alone, the calculated eigenvectors try to model as much variation in the data as possible. Since all samples (dimensions) in such a signal are conterminous due to the temporal relationship, the exhibited variance for each of these dimensions is roughly equal. Situation for this approach is different, since the dimensions in the vector used as input for the analytical transformations have many different sources and value ranges. If there were a feature that is crucial for the recognition of outliers, while at the same time not exhibiting any changes for normal activities, this feature dimension would run the risk of being abandoned, when using dimensionality reduction with the transformation. If the eigenvectors with activity along the mentioned hypothetical feature dimension are removed, the feature dimension is effectively abandoned. Since such an ideal dimension for detecting outliers, however, does not exist, the effect of this will probably be only moderately.

## 5. Experiments

### 5.1. Dataset and Measuring Setup

To be able to compare performance between approaches and configurations, an appropriate metric has to be used. For that, the average class accuracy is used, which is calculated by building the quadratic classification matrix of size |Ω|×|Ω|. Each cell in this classification matrix at row *i* and column *j* represents how many samples of class $\Omega_{i}$ from the dataset have been classified as class $\Omega_{j}$. The diagonal of this matrix thus contains correct classifications, while the surrounding cells represent misclassifications. From this matrix, the average classification accuracy is calculated by averaging over the percentage of correctly classified samples of each class $\Omega_{i}$. To ensure better comparability with other plants, the average F1 score is also given for the most important results. This metric was preferred to weighted F1, because in this use-case it is less important to be right most of the time than to recognize all activities as good as possible, in order to not miss any possible transitions such as stairs. If available, this score is shown in brackets following the average class accuracy. For the final results of each approach, the entire confusion matrix is given in a plot.

Performance evaluations were done using the average class accuracy, on two labeled datasets. For each evaluation, they are randomly split up into training (70%) and test set (30%). Many public domain datasets exist for the pocket-case, such as the MobiAct dataset [56], which was used in this work. This dataset contains accelerometer, gyroscope and orientation data. Since we calculated the orientation from accelerometer and gyroscope using the Madgwick filter, the contained orientation data was not used. In total, the dataset contains recordings from 50 subjects during nine activities, of which four are the activities covered by this work. During the recording of these activities, the smartphone was placed in either the right or the left trouser pocket, at random orientations.

Due to no publicly available datasets for the hand-case, one had to be recorded using our data recording app [57] as demonstrated in [58]. It contains data from eight subjects during the four activities covered by this work, while carrying the device in their hands such that the display is pointing to their face. The dataset was recorded in situations near to reality, such as museum tours. The exact orientation of the smartphone was left to each subjects’ preference, and they were free to change it during the walk.

For this work, both datasets are used in the format of recordings consisting of multiple consecutively executed activities from one person on a pre-defined path, with sensor data sampled at 50Hz. For training, these labeled activity sections that each recording consist of are separately extracted and accumulated per activity class. For training, the activity sequences are extracted from the recordings, and accumulated per activity class. To be able to also correctly handle the transitions between consecutive activities, the sequences are not cut directly at the border between two activities, but instead overlap by $\frac{1}{3}$ of the length of the temporal window used for each approach. Overlapping the extracted activity sequences $\geq \frac{1}{2}$ of the used temporal window’s length does not make sense, since that would lead to the same temporal window existing in two activity classes, which inevitably leads to misclassifications.

The experiments done for each approach are first done separately for pocket-case and hand-case. Performance of the combined dataset with both cases is then examined in Section 5.5. If not further specified, all experiments calculating a classification accuracy use a k-Nearest Neighbor classifier with a neighborhood size of k=3. This is not practical for usage on a mobile device, but was used to avoid the long training times of the SVM, while being very similar in classification performance to a SVM with the RBF kernel [59]. The best performing configuration of each method is then evaluated with an SVM classifier, which establishes the relation to the accuracy that can be expected when run on smartphones.

### 5.2. Results for Analytical Transformations

**Figure 10:**
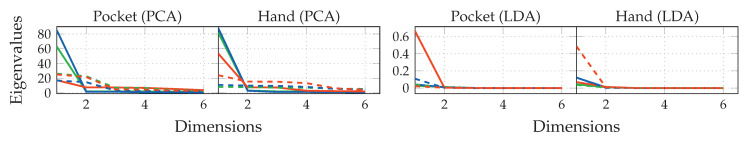
Scree-Plots for a preliminary examination of the information redundancy using both PCA and LDA within a window of length 2 s for pocket-case and hand-case. Green, red, and blue lines represent x, y, and z axes of the accelerometer (solid) and gyroscope (dashed) respectively.

The first experiment for the approach based on analytical transformations is a preliminary examination of the eigenvalues to be expected, when separately training the transformations on hand- and pocket-case dataset, as described in Section 4.1. This was done using a window-length of 100 samples (≊2 s), as suggested by [44,45]. The resulting 10 most significant eigenvalues of both PCA and LDA, when calculated on raw accelerometer (solid lines) and gyroscope (dashed lines) channels, are shown in Figure 10. In case of the PCA, eigenvalues represent the variance along the corresponding eigenvector axes. As discussed in Section 4.1, normalizing the sensor channels’ variances is not necessarily required, but was done here, to allow comparisons between channels. This, however, prevents meaningful comparisons between transformations and cases. From a look at the graphs, a reduction to at most 5 dimensions seems reasonable.

**Figure 11:**
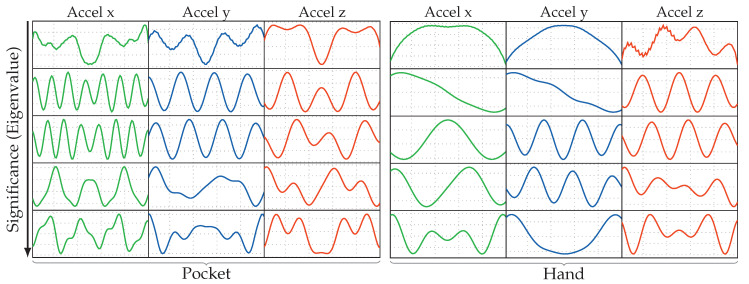
Shapes of the 5 most significant eigenvectors resulting from a PCA calculated on acceleration sequences 2 s in length for both pocket-case and hand-case. Significance decreases from top to bottom.

Next, a quick examination of the eigenvectors trained in the previous experiment was done, to get a look at the basic shapes used to assemble encountered sequences during operation. Figure 11 shows the 5 most significant eigenvectors trained using the PCA for each accelerometer channel and both pocket-case and hand-case. The exhibited shapes look very similar to sine waves with different scaling overall, while some eigenvectors look like a combination of multiple sine waves. More prominent in the pocket-case, some eigenvectors contain irregularities and asymmetries in the vectors. Just like the codewords in a codebook, the eigenvectors contain barely any noise, which suggests that a filter is unnecessary for acceleration. For this setup, the PCA thus behaves similar to a Fourier transform, except that it is not reconstructing signals using simple single sine waves, but combinations of them instead. The eigenvectors exhibited for gyroscope channels look fairly similar and were thus not presented here for a lack of space.

**Figure 12:**
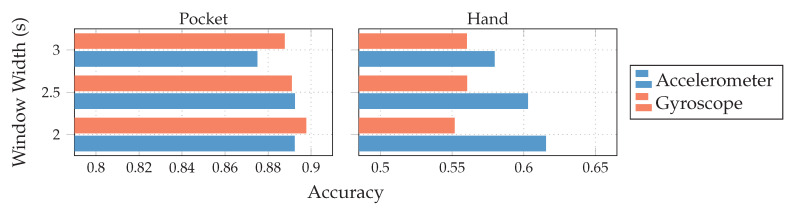
Comparison between classification accuracies when using accelerometer and gyroscope in isolation using a PCA with 5 remaining dimensions.

Both gyroscope and accelerometer were first tested in isolation for pocket-case and hand-case, which helps to determine their roles. In pre-tests, the PCA performed better than the LDA, the tests shown in Figure 12 were thus done with the PCA, and a reduction to 5 dimensions. As can be seen in Figure 12, accelerometer and gyroscope tend to perform similarly in the pocket-case when using 5 dimensions. At a lower dimensionality, however, the gyroscope takes a strong leading role here, which would be in accordance to the discussions from Section 3.2, as well as the findings in [28,29]. Significant is the bad performance of both sensors for the hand-case, with the accelerometer still being better than the gyroscope. This also fits the mentioned discussion above. Differences between the tested window widths of 2 s,2.5 s,3 s, however, were hardly noticeable, with a very light tendency of a decreasing accuracy with higher widths.

**Figure 13:**
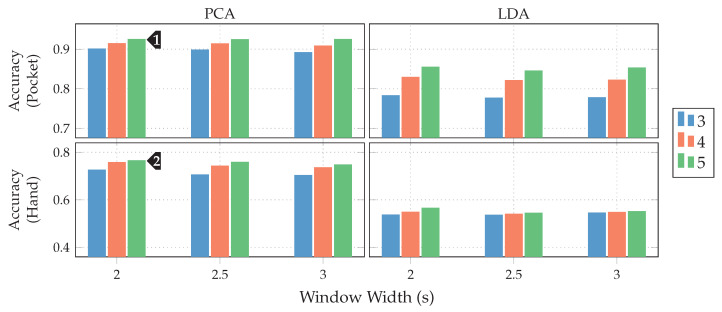
Comparison of the resulting classification accuracies when using the approach based on analytical transformations for different window lengths and dimensions (blue 3, red 4, green 5) with PCA and LDA on both pocket-case and hand-case.

Next, the full approach, including the virtual sensors described in Section 4.1, was run for a set of different window widths, as well as amount of dimensions to which the data is reduced. The results of this test, using both PCA and LDA on pocket-case and hand-case are shown in Figure 13. In this setup, the approach achieved an average classification accuracy of 92.4%(84.1%)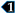 at 5 remaining dimensions with a window length of 2 s for the pocket-case, and 76.4%(80.3%)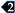 for the hand-case at the same configuration. Between window lengths, again, there is not much difference, with a slight tendency to be better for smaller window lengths. Especially for the hand-case, with more realistic recordings, this could be due to fewer misclassifications at the borders between two adjacent activities. A fundamental question arising from these results is, however, why the LDA performs much worse than the PCA across all tests, even though its primary task is to improve class separability. Since the LDA is especially crafted for classification problems, one would expect it to yield better results than the PCA, which is solely based on variances. Multiple possible reasons for this come to mind. One being that the original Fisher Discriminant Analysis was only specified for two classes. The variant deployed in this work is actually a modification called Multiclass LDA, introduced by Rao in [60]. This variant assumes a normal distribution of the classes, as well as equal class covariances. Another possible reason is that, in comparison to the PCA, the LDA’s kernel is not guaranteed to be positive semi-definite, since it is a combination of multiple covariance matrices. An implication of this are negative eigenvalues, and thus imaginary components in the corresponding eigenvectors. A short test with the trained transformations, however, did not confirm this problem. Another short experiment with training data containing an equal amount of samples per class, instead of the unevenly distributed default, was able to dismiss the distribution of class-samples as another possible implication.

An empirical comparison between PCA and LDA, especially focused on cases that could result in better classification results for the PCA was done in [61]. Sometimes, a chain of LDA and PCA is used for classification problems, where the LDA is used to rotate the data into a coordinate system with better class separability, on which the PCA is then used for dimensionality reduction. A short test with such a chain, however, showed no noticeable improvements. All remaining tests will thus be done with the PCA only.

**Figure 14:**
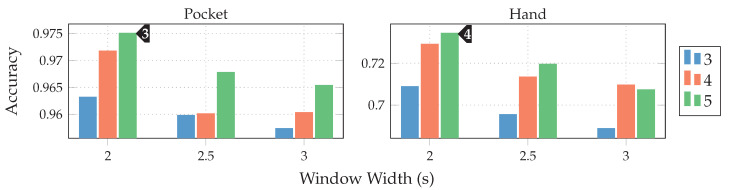
Resulting classification accuracies for the approach based on analytical transformations, with using the PCA at different window lengths and dimensions, on aligned acceleration data in the two-channel coordinate system described in Section 3.1.

As discussed in Section 3.1, the two-channel coordinate system for accelerometer data could help to gain more orientation-independence. The next experiment thus replaced the raw accelerometer with aligned acceleration in this coordinate system, while using the PCA. The aligned acceleration was also used for the calculation of virtual sensors, where applicable. The corresponding results for this experiment are depicted in Figure 14. As can be seen, the best achieved accuracy for the pocket-case was improved from 92.4%(84.1%)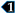 to 97.5%(96.0%)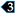 at the same configuration as previously, while the hand-case’s best accuracy has deteriorated from 76.4%(80.3%)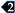 to 73.4%(78.3%)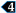. Main motivation for the aligned coordinate systems is an improved orientation-independence, same of which inspired the use of virtual sensors. Acceleration in the two-channel coordinate system could have reduced the information content, such that it is hindering the calculated virtual sensor’s effectiveness. To test this assumption, the comparison between raw and aligned acceleration was repeated without virtual sensors, only making use of accelerometer and gyroscope. And indeed, with hardware sensors only, the two-channel coordinate system was able to improve the best performance from 91.2% to 96.5% for the pocket-case, and from 71.5% to 73.1% for the hand-case.

**Figure 15:**
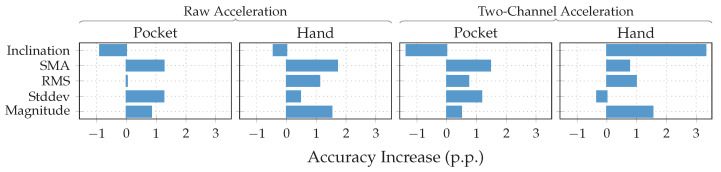
Achieved increases of accuracy ( p.p.) by independently adding each virtual sensor on top of only the hardware sensors, for pocket-case and hand-case, using the PCA with 5 remaining dimensions and a window length of 2 s, using raw acceleration (**left**) and two-channel acceleration (**right**).

To further investigate the virtual sensor’s influence on the approach’s overall classification performance, accelerometer and gyroscope were augmented with each of the virtual sensors individually in the next experiment. The resulting accuracy for each virtual sensor was then compared to the one achieved by the mentioned hardware-sensors alone. This test was repeated with raw acceleration, as well as acceleration in the two-channel coordinate system. As can be seen from the results depicted in Figure 15, most virtual sensors improve the resulting accuracy across all cases. An exception is the inclination sensor, which worsens the result for all cases, except the hand-case using two-channel acceleration. A further investigation of the resulting class accuracies showed that the largest improvements in this case were achieved for the stair activities. The virtual Inclination sensor is the only one, which always uses the raw acceleration for its calculation. Since this sensor brings large gains for the case of the two-channel coordinate system, this could be an indication that the lost information between raw and two-channeled acceleration, probably among other things, is the inclination. Another interesting aspect of this investigation is the finding that the increases in accuracy of the virtual sensors almost add up when combined. Since raw acceleration achieved the better accuracy for the hand-case, it was chosen over the two-channeled acceleration, albeit its inferior performance on the pocket-case.

**Figure 16:**
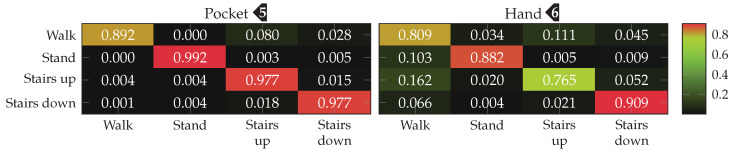
Final confusion matrices for the best-performing configuration (PCA, 5 dimensions, 2 s windows, raw acceleration) of the analytical transformations approach for pocket-case and hand-case using an SVM classifier. Each row represents the actual class, each column the classification results.

Finally, the best performing configuration using a PCA, with a window length of 2 s, 5 remaining dimension and raw acceleration with virtual sensors was tested using the SVM classifier with an RBF kernel. The resulting confusion matrices are depicted in Figure 16. Compared to the accuracies achieved with the Nearest-Neighbor classifier for this configuration, the SVM classifier was able to improve them from 92.4%(84.1%)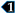 to 96.0%(89.3%)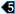 for the pocket-case, and from 76.4%(80.3%)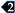 to 84.1%(73.9%)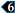 in the hand-case. This improvement can probably be mostly attributed to the small neighborhood size of k=3, which was chosen for performance reasons. As was to be expected from the discussion in Section 3.2, most misclassifications in the hand-case happen between the gait-involving activities.

Since the SVM classifier significantly improved accuracies for raw acceleration, it was also tested with the two-channel coordinate system for acceleration. One advantage of this coordinate system, especially for the mobile use-case, is its reduced dimensionality, which causes a lower overall computational complexity for the approach. In this setup, the approach was able to achieve 96.1% and 83.0% for pocket-case and hand-case respectively. The two-channel configuration of this approach could thus be a worthwhile tradeoff, when considering the minor difference in accuracy.

#### Outlier Detection

Next, the approach’s generated features are tested for outlier detection. For that, a one-class SVM with the RBF kernel was trained on a combination of the datasets for hand-case and pocket-case. To determine the classification accuracy, a part of the combined dataset was split for testing as positive class samples. To test the class accuracy for the detection of outliers, the mentioned unrepresentative dataset of people messing around with their smartphones was used. A gradient descent approach was then used to find an appropriate scaling parameter γ for the RBF kernel that results in best classification accuracies across both negative and positive samples. A classification accuracy of up to 95.5% was achieved, for γ=0.005. At this best configuration, the outlier detection was able to correctly classify 94.1% of the positive samples, and 97.0% of the negative samples.

### 5.3. Results for Codebook

Next, the approach based on codebooks is evaluated. Not all of the approach’s numerous hyperparameters will be evaluated in this section. The parameter $n_{\mathrm{sub}}$, which controls the amount of extracted subsequences per sequence, was set to $n_{\mathrm{sub}}=32$, which was selected in a pre-test. The length of these subsequences is controlled by $w_{\mathrm{sub}}$. Values for this hyperparameter are evaluated, since it has shown to have a higher influence on the achieved classification accuracies than $n_{\mathrm{sub}}$. The hyperparameter $s_{i}$ controls the step width used for the extraction of subsequences from a sequence, which has been empirically selected as $s_{i}=2$. From these hyperparameters, the actual length $w_{\mathrm{seq}}$ of the extracted sequences can then be determined using (6). The remaining evaluated hyperparameter are σ, which controls the assignment smoothness of the Kernel Density Estimation, and |C|, which controls the number of codewords per codebook. As before, if not explicitly stated otherwise, all benchmarks are done using a k-Nearest Neighbor classifier with a neighborhood size of k=3.

As a first test for these hyperparameters, the approach based on codebooks, as described in Section 4.2, was run with different combinations of values for the hyperparameters σ, $w_{\mathrm{sub}}$, and |C|. Since it was using raw accelerometer and gyroscope data, two codebooks were used. The tested values for $w_{\mathrm{sub}}$ were chosen, such that they roughly represent the range between 2 s and 3 s for the overall sequence lengths, where the number of subsequences per sequence $n_{\mathrm{sub}}$ is fixed to 32 as mentioned above. Thus, the subsequence lengths 32≈1.88 s, 48≈2.2 s, 64≈2.52 s, and 96≈3.16 s were tested. This test was repeated for both assignment strategies that allow using a codebook for multiple sensor channels. The strategy that appends subsequences before assigning them through the codebook achieved accuracies which were lower by roughly 2 percentage points when averaged across all tested configurations. The remaining evaluation will thus focus on the strategy that treats all subsequences independently.

**Figure 17:**
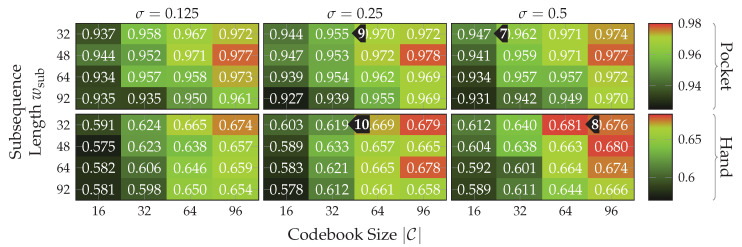
Classification accuracies achieved with the approach based on codebooks for multiple different combinations of the σ, $w_{\mathrm{sub}}$, and |C| hyperparameters for both pocket-case and hand-case.

The results depicted in Figure 17 show the accuracies achieved for pocket-case and hand-case at different assignment smoothnesses σ, codebook sizes |C|, and subsequence lengths $w_{\mathrm{sub}}$. Each tested assignment smoothness σ is represented as one heatmap of accuracies. The tested values were adopted from [19] for an initial pre-test, but turned out to already be in the optimal range for dataset and use-case at hand. While there is not much change in classification accuracy between them, using lower or higher values only decreased accuracy for both pocket-case and hand-case. Considering the resulting classification accuracy as a function of codebook size |C| and subsequence length $w_{\mathrm{sub}}$ reveals a similar pattern across tested σ. As was to be expected, an increasing |C| leads to higher accuracies, while already demonstrating surprisingly good accuracies of up to 94.7%(94.3%)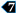, with only 16 codewords for the pocket-case. Further increases in classification accuracy would not be worth the increased computational complexity, for the pocket-case at least. The sweet spot for the used subsequence length here seems to be at 48 samples. With the highest classification accuracy of 68.1%(72.8%)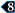 for the hand-case at σ=0.5, $w_{\mathrm{sub}}=32$, and |C|=64, however, the accuracies are mostly insufficient for practical uses. Improvements of the classification accuracy achieved by increasing the number of codewords in a codebook, is 3 percentage points on average, over the tested codebook sizes. This improvement is clearly more significant than the one observed for the pocket-case above, but still only marginal for practical uses. The sweet spot for the subsequence length $w_{\mathrm{sub}}$ seems to be somewhere between 32 samples and 48 samples, instead of 48 observed for the pocket-case results. This may be connected with the main difference between pocket-case data and hand-case data, which was discussed in Section 3.2. Since the smartphone can only measure movements of the leg to which the pocket is attached, the exhibited base frequency in the data recorded for the pocket-case is roughly half that of the hand-case. Most repetitive behavior in the recorded signals for the hand-case thus have smaller periods, which could be advantaged by a smaller subsequence length $w_{\mathrm{sub}}$.

To test the contribution of accelerometer and gyroscope to the results achieved in the tests above, classification was next tested separately on both sensors. For this test, a subsequence length $w_{\mathrm{sub}}$ of 32 was used, since that tends to perform best for the hand-case, while still performing reasonably well for the pocket-case. With codebooks of size |C|=32 and σ=0.25, this test yielded an accuracy of 90.7% and 92.4% on the pocket-case dataset for accelerometer and gyroscope respectively, compared to 95.5%(95.6%) 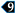 with both sensors in combination. While the gyroscope does seem to be more suited for the recognition of pocket-case data, it is only a marginal lead.+ Since the combined accuracy is higher, the accelerometer does seem to still have valuable information to contribute. On the hand-case dataset, the accuracies 63.19% and 50.36% were achieved for accelerometer and gyroscope respectively. This can be explained by the exhibited shapes in the analysis done in Section 3.2. It makes sense that the accelerometer is taking the leading role for the hand-case, since, as discussed in the mentioned section, barely any characteristic rotations should reach the smartphone when carried in hand. With a classification accuracy of 61.9%(66.2%)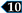, with the combination of accelerometer and gyroscope it seems that the gyroscope is actually obstructive for classification.

**Figure 18:**
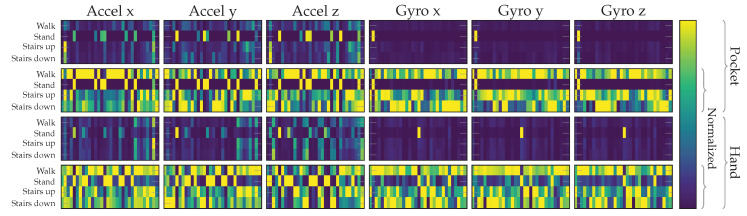
Heatmaps demonstrating the average codeword assignment for accelerometer and gyroscope data of pocket-case and hand-case, on a codebook of size 32 broken down by activity. The upper row for each case shows the unnormalized assignment averages, while the respective second lines were normalized per codeword (per column, across all activities). This allows to see tendential differences between activity-specific assignments, without losing sight of the real significance of differences.

The codebook approach can offer interesting insights into the similarity of observed shapes in the data of all covered activities. For this investigation, the constructed accelerometer and gyroscope codebooks from the previous experiment are used to assign the complete set of testing samples from the datset. Calculating the average over all assignment histograms and displaying them in a matrix or a stack of histograms then reveals the most and least strongly assigned codewords in a codebook, broken down by class. These matrix plots for pocket-case and hand-case are shown in Figure 18. Each of these matrix plots is contained twice, with and without normalization. While the unnormalized plots visualize the actual results and facilitate comparisons, the plots with normalization, done per codeword (column) across all activities, allows for a simple mapping of which codeword is used most by which activity. The normalized plot matrices show that the exhibited patterns for each activity seem to use different codewords, which hints at the separability of activities. The differences between codewords used for different activities does seem to be slightly more pronounced for the pocket-case than for the hand-case, meaning that codewords used for an activity are less active in others. However, when compared with the unnormalized plots, it becomes clear that the normalized plots exaggerate the difference between activities, while the actual difference is much less significant. As can be seen in the unnormalized plots, especially the accelerometer’s x-axis and y-axis exhibit a very similar assignment pattern across all gait-based activities. The assumption of a difficult distinction between the activities involving gait based on the exhibited shapes in the hand-case from Section 3.2 thus proved valid.

**Figure 19:**
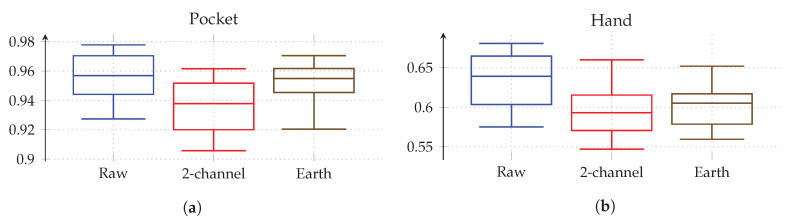
Statistical evaluation of the accuracies across all tested configurations for raw acceleration, two-channel acceleration, and acceleration transformed into the underspecified earth coordinate system for both pocket-case (**a**) and hand-case (**b**).

As for the previous approach, the next test evaluates performance with aligned coordinate systems for accelerometer data. Each of the two aligned coordinate systems was tested for all previously benchmarked hyperparameter configurations on the raw accelerometer data. The statistical evaluation of the resulting classification accuracies is shown in Figure 19 for pocket-case and hand-case. As can be seen in the box plots, the codebook approach performed worse on the aligned coordinate systems when compared to raw acceleration. So the codebook seems to be able to get more information from the raw data, than from the artificially transformed data. A reason for this might be that the two-channel coordinate system deliberately discards information by reducing the two remaining axes aside of the gravitational axis, to their magnitude value. If the codebook is able to extract valuable information from all three raw sensor channels, this combination into the magnitude might be discarding valuable information, thus resulting in lower accuracies, which would explain why the underspecified earth coordinate system performs worse than raw, but still better than two-channel acceleration. All remaining tests will thus remain using raw acceleration instead of an aligned coordinate system.

**Figure 20:**
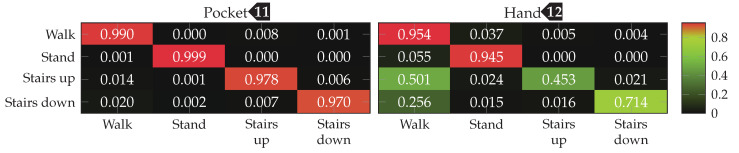
Final confusion matrices for the best-performing configuration (σ=0.25, |C|=32, $w_{\mathrm{sub}}=32$) of the approach based on codebooks for both pocket-case and hand-case using an SVM classifier. Each row represents the actual class, and each column represents the classification results.

The accuracy improvements observed between the tested configurations were not significant enough, to justify the increase in computational complexity for the better performing configurations. For the test with an SVM classifier, thus σ=0.25, and |C|=32 was chosen as base configuration. For $w_{\mathrm{sub}}$, the two values 48 and 32 were tested, as the two observed sweet spots for pocket-case and hand-case. With subsequences of length $w_{\mathrm{sub}}=48$, the k-Nearest Neighbor classifier achieved accuracies of 95.3% and 63.3% for pocket-case and hand-case respectively. When the same configuration is tested using the SVM classifier, the classification accuracies of 98.1% for the pocket-case, and 73.7% for the hand-case are achieved. This is a small difference for the pocket-case, but a significant difference for the hand-case. Like for the approach based on analytical transformation, part of this difference can probably be attributed to k=3 as the chosen neighborhood size for the k-Nearest Neighbor classifier. With $w_{\mathrm{sub}}=32$, the SVM classifier performed slightly better, achieving classification accuracies of 98.4%(98.1%)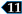 and 76.6%(81.6%)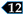 for pocket-case and hand-case respectively. The minor improvement of accuracy for the pocket-case can be mostly assigned to measurement errors. Nevertheless, the classification shows an accuracy for the pocket-case, which is at least on par with the classification for $w_{\mathrm{sub}}=48$, even though the sweet spot for the pocket-case seemed to be around the latter from the previous benchmark results. With $w_{\mathrm{sub}}=32$ and the k-Nearest Neighbor classifier, this configuration previously achieved the classification accuracies 95.5%(95.6%) 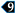 and 61.9%(66.2%)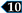 for pocket-case and hand-case respectively. The resulting confusion matrices when using the SVM classifier with $w_{\mathrm{sub}}=32$ for pocket-case and hand-case, are shown in Figure 20. To no surprise, the most common misclassifications are found between the activities that involve gait.

#### Outlier Detection

Finally, the approach’s generated features are tested for outlier detection. Identical to the previous outlier test, a one-class SVM using an RBF kernel, was trained on a subset of the features calculated on the combined dataset from pocket-case and hand-case, using the previously best performing configuration. Accuracy was tested with the complementary subset of the combined dataset, as well as the unrepresentative dataset for people messing around with their smartphone. To find an appropriate kernel scaling parameter γ that results in best classification accuracies across both negative and positive samples, a gradient descent approach was used. The tested configuration uses an assignment smoothness of σ=0.25, a subsequence length of $w_{\mathrm{sub}}=32$, and a codebook size of |C|=32. The best classification accuracy reached with this configuration is 86.0%, at a scaling parameter of γ=15.0 for the RBF kernel. Accuracy of the positive class was 73.1%, and that of the negative class was 99.0%. Due to the low accuracy for the positive class, the outlier classification accuracy on pocket-case and hand-case was tested in isolation, using γ=15.0. With 92.3%, this test surfaced a high accuracy for the pocket-case, and a low accuracy of only 71.0% for the hand-case. This probably was to be expected, due to the approach’s overall difficulties for the hand-case dataset. Executing the hyperparameter optimization for γ on the hand-case dataset alone, resulted in an accuracy of up to 85.7% at γ=11.1. This corresponds to an accuracy of 77.0% for the positive class, and an accuracy of 94.5% for the negative class. The concerns discussed in Section 4.2 regarding outlier detection with features from the codebook approach thus seem valid. Since the codebook is not trained with outlier sequences, there are probably no codewords correctly representing these cases, leading to ambiguous feature vectors.

### 5.4. Results for Statistical Features

This section will focus on evaluating performance characteristics of the approach based on statistical features, as introduced in Section 4.3. Since both MFCC and AR-Coefficient features themselves have hyperparameters, we first searched for the best performing configuration for both. To exclude the influence of other features in these tests, both were tested in isolation to find suitable values for these hyperparameters. For that, the approach’s pattern recognition pipeline, as visualized in Figure 8, was employed, while only calculating these individual features as part of the feature extraction step. As before, if not explicitly stated otherwise, all benchmarks use a k-Nearest Neighbor classifier with a neighborhood of k=3.

**Figure 21:**
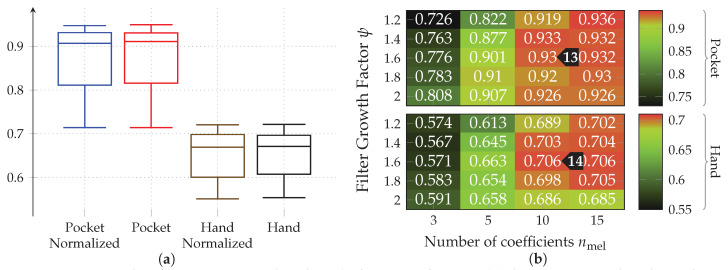
Results of hyperparameter benchmarks for MFCC features. (**a**) shows a statistical analysis of the accuracy distributions for different hyperparameters, dependent on case and whether normalization was used for the filter-bank. (**b**) shows the accuracies reached as a function of hyperparameters $n_{\mathrm{mel}}$ and ψ, averaged across the tested window lengths for both pocket-case and hand-case.

In case of the MFCC features, tested hyperparameters are $n_{\mathrm{mel}}$ and ψ, which control the number, as well as the growth of filters used in the underlying filter-bank respectively. For $n_{\mathrm{mel}}$, the values {3,5,10,15} were tested, while the values for ψ ranged from 1.2 to 2.0, using steps of 0.1. After a short pre-test, the analytical transformation LDA with a reduction to 4 dimensions was chosen. The amount of 4 dimensions while using only either MFCC or AR-Coefficients as feature demonstrated to preserve most of the information, while allowing a much faster classification. As listed in Table 2 in Section 4.3, MFCC features are calculated on 8 channels (accelerometer, gyroscope, accelerometer magnitude, gyroscope magnitude). For $n_{\mathrm{mel}}=15$, this already amounts to 120 features, which is clearly reflected in the speed of classification without the analytical transformation. These testes were done twice, with, and without normalization of the filters in the filter-bank. Statistical evaluation of the resulting accuracies for pocket-case and hand-case with enabled and disabled normalization of the filters is shown in Figure 21a. As can be seen, there is barely any difference to be found between the performance of both normalized filter-banks and filter-banks without normalization. For the pocket-case, the highest accuracies over all tested parameters with normalization (94.8%) and without normalization (94.9%) can be assigned to measurement errors. Same goes for the hand-case, where the best accuracies are 72.1% and 72.2% with and without normalization respectively. The remaining benchmarks will use MFCC features with unnormalized filter-banks.

**Figure 22:**
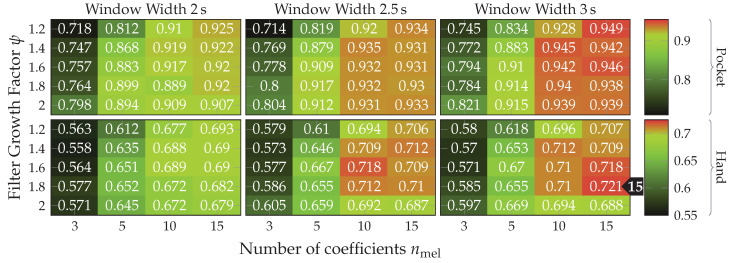
Results of benchmarks done for the hyperparameters $n_{\mathrm{mel}}$ and ψ, controlling number, as well as growth of filters in the used MFCC filter-bank respectively, for both pocket-case and hand-case.

Next, the accuracies of the benchmark using unnormalized filter-banks will be evaluated with respect to the tested hyperparameter configurations. Figure 21b shows the reached accuracies, averaged across the tested window lengths, as a function of hyperparameters $n_{\mathrm{mel}}$ and ψ for both pocket-case and hand-case. As can be seen, the best performing configuration across cases is $n_{\mathrm{mel}}=10$ and ψ=1.6, which reaches an average accuracy of 93.0%(94.0%)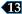 for the pocket-case, and 70.6%(72.8%)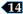 for the hand-case. The benchmark results for the tested window lengths can be seen in Figure 22. The patterns exhibited across the tested window lengths, as well as across cases are very similar. Here, the improvements for accuracy, reached by increasing the number of calculated MFC coefficients $n_{\mathrm{mel}}$, declines with each step. The average difference between $n_{\mathrm{mel}}=3$ and $n_{\mathrm{mel}}=5$ roughly equals 11.5 p.p. for the pocket-case, and 7.5 p.p. for the hand-case. Despite being a larger step, increases between $n_{\mathrm{mel}}=5$ and $n_{\mathrm{mel}}=10$ only amount to 4.1 p.p., and 4.8 p.p. for pocket-case and hand-case respectively. If the accuracies achieved are considered to be a function of the tested growth factors ψ, only minor differences are to be found for $n_{\mathrm{mel}}\geq 10$. The greatest influence of the tested growth factors can be seen for $n_{\mathrm{mel}}<10$. Here, the accuracy is better, the larger the growth factors are chosen. This can be interpreted as an indication of the importance of low frequencies, as larger growth factors lead to more and smaller filters in the lower frequency ranges. This causes the calculated feature’s to have a higher resolution in the lower frequency ranges. Due to the relatively slow nature of gait, this seems plausible.

**Figure 23:**
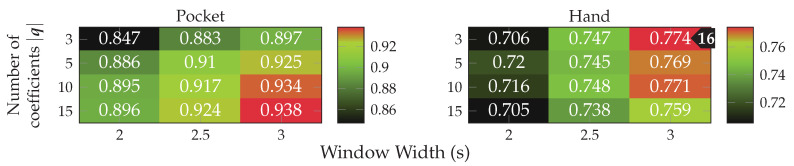
Resulting classification accuracies for the approach based on statistical features, when only using AR-Coefficients at different hyperparameter configurations, shown as a function of the model order |q| and the used window length for both pocket-case and hand-case.

Next, a benchmark for the hyperparameter |q| of AR-Coefficient feature calculation was conducted. This hyperparameter, controls the amount of coefficients that are calculated per sequence. The results for this benchmark are visualized in Figure 23. As can be seen, the AR-Coefficients tend to perform better with larger windows. When comparing the achieved accuracies between windows of length 2 s and 3 s, the average difference for the pocket-case is 4 p.p., while the average difference for the hand-case is 5.25 p.p.. The biggest improvement for both cases is here achieved for a model order of |q|=3. When examining the influence of |q| on the achieved accuracies, only a small influence can be detected. By increasing |q| from 3 coefficients to 15 coefficients, only an average improvement of 4.33 p.p. can be achieved in the pocket-case. The highest improvement for the pocket-case is seen between |q|=3 to |q|=5. In the hand-case, the hyperparameter does not seem to have any significant influence. Accuracy even tends to decline with a growing model order |q|. This may be due to differences in the complexity of the signals between pocket-case and hand-case. Such a tendency can also be observed when comparing the eigenvector’s shapes, calculated as part of the analytical transformation-based approach’s evaluation, shown in Figure 11. When comparing the exhibited shapes for the accelerometer, the eigenvectors for the pocket-case seem to contain slightly more irregularities and assymetries than the shapes exhibited for the hand-case. Though, it should be noted here that shapes which look like combinations of multiple sine waves can be found for both cases equally. For the pocket-case, however, these shapes can be found across all eigenvectors, while for the hand-case, the more complex shapes tend to occur in eigenvectors of lower significance. So, a model order of |q|=3 seems to be enough to model the exhibited shapes for the hand-case. The weak tendency of a declining accuracy for a growing model order for this case could thus be credited to the AR-Coefficients starting to model the noise in the signal with a larger number of coefficients. As best compromise for both cases, thus a value of 5 is chosen for the hyperparameter |q|.

When comparing accuracies for the hand-case between AR-Coefficient (max 77.4%(81.3%)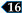 ) and MFCC features (max 72.1%(74.0%)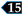 ), the AR-Coefficients prove to be superior. Situation for the pocket-case is more complex. Here, the AR-Coefficients tend to perform better than the MFCC coefficients when only calculating 3 coefficients for both approaches, reducing in performance for a larger model order |q|. To summarize, AR-Coefficients seem to be more efficient by producing higher accuracies for smaller numbers of coefficients, and thus smaller feature vectors. In addition, AR-Coefficients also have the advantage of being dependent on only one hyperparameter, while MFCC features must also be adjusted to each use-case, by tuning the filter growth factor ψ for their underlying filter-bank.

**Figure 24:**
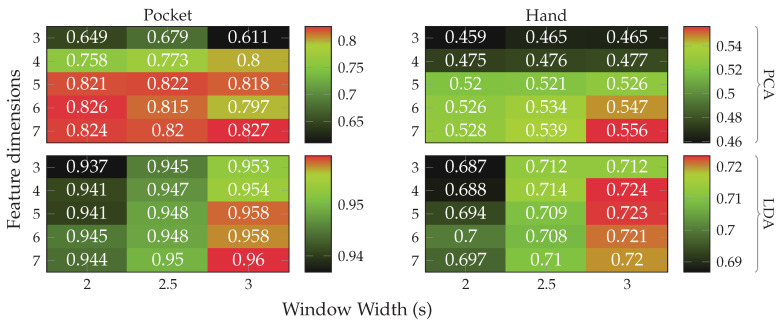
Classification accuracies achieved for different window widths and remaining dimensions after the analytical transformation on the concatenated feature vector. The benchmark was done for both pocket-case and hand-case, comparing the use of both PCA and LDA.

After the most efficient hyperparameter configuration for both MFCC and AR-Coefficients have been found at $n_{\mathrm{mel}}=10$, ψ=1.6, and |q|=5, next, the remaining benchmarks using the full feature set are carried out using these values. For the dimensionality of the feature vector after the analytical transformation, this benchmark tests the value range [3,7]. Again, the window lengths 2 s, 2.5 s, and 3 s, as well as both analytical transformations PCA and LDA are tested. The benchmark results for pocket-case and hand-case with PCA and LDA can be seen in Figure 24. As can be seen, for this approach, the LDA is generally faring much better than the PCA. However, even with the LDA, using the full set of features barely brought any improvements over the classification accuracy achieved using MFCC and AR-Coefficient features in isolation. The trend of better classification accuracies for larger window lengths, however, is analogous to the one observed for the trend observed in the isolated tests with the MFCC and AR-Coefficients above. All remaining tests will thus focus on the LDA.

**Figure 25:**
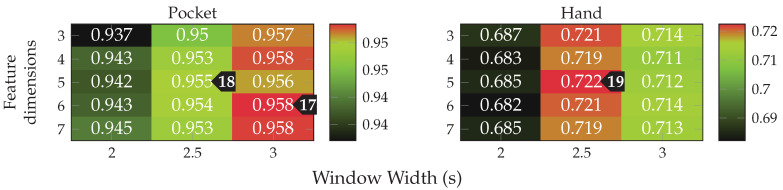
Classification accuracies achieved for different window widths and remaining dimensions after the LDA, while using two-channel acceleration for both pocket-case and hand-case.

Next, the approach was tested with the aligned coordinate systems for accelerometer data. Results of the two-channel coordinate system are shown in Figure 25. When compared to the classification accuracies reached with raw sensor data, a minor improvement was achieved for the hand-case, while the performance on the pocket-case degraded by the same amount. The amount of features to calculate before compacting them by applying the analytical transformation, however, is smaller for the two-channel coordinate system. Thus, the combination of better classification accuracies for the hand-case, as well as an improved performance is welcome. Additionally to these advantages, the sweet spot for the used window length for the hand-case has moved from 3 s to 2.5 s, which reduces the approach’s overall delay. With 95.8%(94.7%)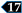, the best accuracy for the pocket-case here is reached for 6 remaining dimensions and a window of 3 s. The hand-case achieved a maximum classification accuracy of 72.2%(73.6%)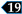 at 5 remaining dimensions and a window of 2.5 s. Like when using unaligned acceleration, this result is much worse for the hand-case when compared to using only AR-Coefficients 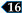. The assumption is therefore that some of the included features confuse the classifier, even though the LDA acts as a kind of simple feature selector when paired with a reduction in dimensionality. Acceleration in the two-channel acceleration was chosen as best configuration, since it achieves similar performance to unaligned acceleration at smaller window sizes, thus reducing delay while also operating with fewer dimensions.

**Figure 26:**
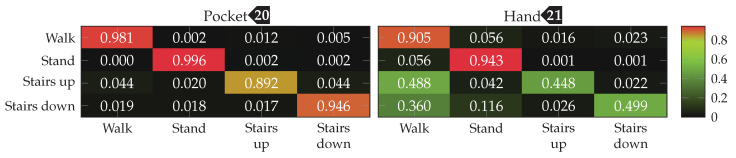
Final confusion matrices for the best-performing configuration (LDA, 2.5 s windows, 5 dimensions, two-channel acceleration) of the statistical features approach, for pocket-case and hand-case using an SVM classifier. Each row represents the actual class, each column the classification.

Finally, the configuration which performs best across both cases is tested using an SVM classifier with the RBF kernel. The tested configuration uses a window length of 2.5 s, a feature vector with 5 dimensions after the LDA and is calculated using accelerometer data in the two-channel coordinate system. With the k-Nearest Neighbor classifier, this configuration previously achieved 95.5%(94.7%)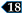 and 72.2%(73.6%)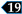 for pocket-case and hand-case respectively. When using the SVM classifier, this configuration achieves 95.4%(94.7%)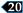 and 69.9%(73.0%)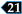, with the corresponding confusion matrices depicted in Figure 26. The difference for the pocket-case can here be assigned to measuring errors, while the result for the hand-case with the SVM changes little for the low overall practicability of the approach. For comparison, using only AR-Coefficients as feature with an SVM classifier, a window length of 2.5 s and 4 dimensions after the LDA, achieved the classification accuracies 89.6% and 74.9% for pocket-case and hand-case respectively.

#### Outlier Detection

Finally, the approach’s generated features are tested for outlier detection. This procedure was identical to the outlier detection tests done for the two other approaches. A one-class SVM using the RBF kernel was trained on a subset of the features calculated on the combined dataset from pocket-case and hand-case. Accuracy was tested with features from the complementary subset, as well as the unrepresentative dataset for people messing around with their smartphone. To find an appropriate kernel scaling parameter γ that results in best classification accuracies across both negative and positive samples, a gradient descent approach was used. For this investigation, the best performing hyperparameter configuration, as determined by the previous benchmarks, was used. At a window length of 2.5 s and 5 remaining dimensions after reduction by the LDA, this configuration uses AR-Coefficients with a model order of |q|=5, and calculates $n_{\mathrm{mel}}=10$ MFC coefficients per sensor channel, at a filter growth of ψ=1.6 for an unnormalized filter-bank. Using this setup, the best classification accuracy for outlier detection achieved was 92.4% at a γ=4.55. This classification accuracy is composed of the class accuracies 95.8% for the positive, and 89.1% for the outlier class.

### 5.5. Comparison and Discussion

Finally, we try to provide a brief comparison of the performance characteristics exhibited between the three tested techniques. An extensive list of the achieved accuracies of other approaches, run on the MobiAct data set used for the pocket-case, can be found in [62]. The average accuracy calculated across all of the 18 listed works with at least our set of recognized activities, is 85.54% with a standard deviation of ±10.98. An overview of our resulting classification accuracies for the hyperparameter configurations determined to be the most efficient, tested with both k-Nearest Neighbor and SVM classifiers, are shown in Table 3. As can be seen from the listed numbers, the overall best technique seems to be the approach based on analytical transformations, introduced in Section 4.1. It performed best in combination with unfiltered acceleration in the device’s local coordinate system. However, if an SVM classifier is used, the difference in accuracy to two-channel acceleration is relatively small, while the computational complexity is noticeably smaller. Using two-channeled acceleration might thus be a good tradeoff to make, when employing the approach on a mobile device. When compared with the other approaches, it achieves the best classification accuracies for outlier detection, and hand-case, while only being in measurement error range from the best technique for the pocket-case. With a classification accuracy of roughly 84% when only trained for the hand-case, this approach is the only one above 80%. For practicability reasons, this approach will thus be the only one tested on a combined dataset including pocket-case and hand-case below.

For the pocket-case, the technique based on codebooks, introduced in Section 4.2, achieved classification accuracies on the level of the ones achieved by the analytical transformation-based approach. However, when comparing the accuracies for the hand-case achieved with the k-Nearest Neighbor, it exhibits the worst classification accuracy by a large margin. Switching to the SVM classifier brought surprising improvements here. As mentioned in Section 5.3, this large difference can at least partly be attributed to the chosen neighborhood size of k=3, which was mostly used for performance reasons with benchmarks using the k-Nearest Neighbor classifier. Only raising the codebook sizes to disproportionately large numbers was able to improve the classification accuracy for the hand-case. Especially for mobile use-cases, the resulting computational complexity here would quickly be impractical. Tests with aligned acceleration showed no improvements with the codebook technique. For outlier detection, the codebook-based approach proved to be the least performing. This is in correspondence with the assumptions and possible underlying problems of codebooks discussed in Section 4.2. Due to the unsatisfactory accuracy for the hand-case, as well as the poor accuracy for outlier detection, we consider the approach unsuitable for the practical use in the combined case containing pocket and hand data.

As might be expected from the numerous studies using similar methods, the technique based on statistical features, described in Section 4.3, performed well on the pocket-case. Performance on the hand-case, when tested using an SVM classifier, however, is far behind the other tested approaches. Nevertheless, when comparing the resulting classification accuracy for outlier detection, it is able to compete with the one based on analytical transformations. Despite its good performance for the pocket-case and outlier detection, the poor accuracy achieved in the hand-case makes this approach unsuitable for the practical use with both cases combined. Though, as we noted in the evaluation, our version of the approach certainly has a feature selection problem, and can thus not generally be dismissed for satisfactorily solving the hand-case.

When the approaches based on codebooks and analytical transformations were tested with accelerometer and gyroscope in isolation, they exhibited very similar behavior. In correspondence with the discussion in Section 3.2, the accelerometer is performing better than the gyroscope for the hand-case. The difference between both sensors, though, was unexpectedly small for both approaches. For the hand-case, there thus apparently is more information to be found from the gyroscope than expected. This assumption is further supported by the test results with acceleration in the two-channel coordinate system. Both mentioned approaches deliver worse results with two-channel acceleration. However, with the analytical transformations-based technique, the virtual Inclination sensor as described in Section 4.1, brought back some of the accuracy that was lost when switching to the aligned coordinate system. This can be interpreted as an indication that the temporal progression of a smartphone’s orientation is a valuable source of information for the hand-case.

**Figure 27:**
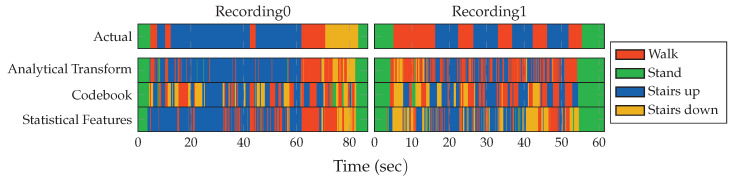
Classification results for two particularly badly classified recordings between all of the three presented procedures in comparison with the actually performed activities (as shown in the first row).

Figure 27 compares the classification results of all three described approaches with the actually performed activities for two particularly badly classified recordings. Both are recordings for the hand-case, and were not part of the training dataset. All of the approaches were employed in their best configuration, while using the SVM classifier, as shown in Table 3. For Recording0, our novel approach based on analytical transformations performed best. It gets most of the stairs up activity right, though also exhibits many small variations. Compared to the codebook approach, however, our method does not recognize the relatively short walk sequence in the middle. The reason for this could be the decomposition of sequences into subsequences for the Codebook approach, since the first subsequences start matching comparably fast on the start of new activity sequences. For this recording, the technique based on statistical features generally has problems detecting the stairs up activity.

Recording1 shows the walk on stairs with large plateaus between the stair segments. Here, all approaches perform poorly, which is expressed by strong fluctuations of the detected activity in the graph. However, while our novel approach mainly cannot decide between walking and climbing stairs, the other two approaches also show misclassifications for the stair descend activity. This large number of fluctuations is mainly due to the boolean decision for only one class using SVMs. When instead training a set of SVMs for class probabilities, these decisions are less pronounced [63]. For both recordings, these observations coincide with the general problems of distinguishability between locomotion activities in the hand-case, which were discussed in Section 3.

**Figure 28:**
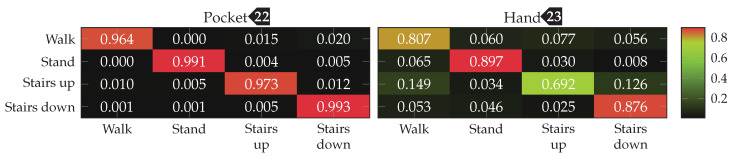
Confusion matrices for both pocket-case and hand-case, when evaluated separately on a model trained on the combination of both datasets. As model, the approach based on analytical transformations with a PCA, 5 remaining dimensions, a window length of 2 s, raw acceleration, and an SVM classifier with the RBF kernel was used.

Finally, the analytical transformations-based technique was tested on the combined dataset, containing data for both pocket-case and hand-case. This test was done with the configuration shown in Table 3, while using an SVM classifier with the RBF kernel. The subset used for training was chosen evenly distributed, such that all activities had a representative set of samples with equivalent size. The overall accuracy was improved by including slightly more samples for the hand-case, than for the pocket-case. After the model has been trained with this combined training dataset, the both cases were evaluated independently. The resulting confusion matrices for both cases are shown in Figure 28. With 98.0%(94.9%)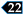, the model was able to preserve its high classification accuracy for the pocket-case of previously 96.0%(89.3%) 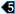. For the hand-case, the classification accuracy dropped to 81.8%(74.0%)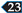 from the 84.1%(73.9%) 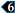 achieved with separate training on both cases. When comparing the results with the separately trained counterpart depicted in Figure 26, the walking upstairs activity can be identified as main loser of this combination for the hand-case. The loss in accuracy incurred by the combined training is less pronounced than expected. Though with roughly 80% for the hand-case, the accuracy still has room for improvement.

## 6. Conclusions

In this paper, we described the use of activity recognition in an indoor localization use-case, which includes both carrying the smartphone in hand (hand-case), or in a trouser pocket (pocket-case). To the best of our knowledge and conscience, the hand-case has not yet been examined with practical applicability in mind by any publication, which makes it a novel use-case. We then introduced and tested three possible approaches separately on pocket-case and hand-case datasets. For reasons of practical applicability, we have limited the choice of sensors to accelerometer and gyroscope. The magnetometer sensor was examined by existing research [28,29], and showed no sign of improving accuracy, when used additionally to accelerometer and gyroscope. The barometer, which is very promising for the detection of changes in altitude, was excluded as it is not yet widely used in common smartphones.

The tested techniques are a mixture of novel and adapted concepts. The analytical transformation-based approach was not found in other studies, and thus introduced as novel activity recognition technique. It uses a sliding window to extract sequences from each sensor channel, and uses a unique analytical transformation per such channel, to reduce dimensionality of the extracted sequences. This technique has been further extended by the concept of virtual sensors, which calculate new virtual sensor channels by computing a selected set of metrics on the raw sensor data of gyroscope and accelerometer. The second introduced approach is based on codebooks, and was adapted from [19], where it was originally introduced for activity recognition with wearable sensors. It is a feature learning approach that tries to learn unique and significant sequences identifying the activities. Since almost every publication in this research area considers an unidentical set of activities, a fair comparison of the accuracies is problematic. Thus, we chose an approach based on statistical features as third approach, which is the one used by most research in the smartphone activity recognition field. This way, the resulting accuracies of this technique can be seen as the comparison to most existing research, though limited to the classifiers we used.

The approach based on analytical transformations, as best performing in the comparison of pocket-case and hand-case trained and tested separately, was finally evaluated on the combined dataset containing the sequences from both cases. To gain more insight on the performance, the training sequences of both cases were then tested separately, to obtain comparable accuracy metrics for both cases. When the sequences used for training were carefully balanced between both cases, the resulting accuracies of 98.0% for pocket-case and 81.8% for the hand-case barely changed compared to the previously models separately trained on both cases. As expected from the discussion at the beginning of this paper, most of the misclassifications can be found between the gait-involving activites. With activity recognition in the supporting role of indoor localization, an accuracy of roughly 80% is acceptable, but has room for improvement.

### Future Work

For example, the integration of a light or proximity sensor could be a valuable data point, contributing to the differentiation between pocket-case and hand-case, thus potentially improving accuracy for the combined version. However, also in isolation, achieving good classification accuracies for the hand-case, which was considered in this work, proved to be difficult. A thorough investigation of existing research has shown that this case has not yet been part of many investigations. The few publications with a similar case all made use of a barometer sensor for the detection of ascend and descent. The most similar sensor position with wide coverage in existing research, are wrist-worn wearables. This position generally works much better than the hand-case, since these devices are more similar to a wristwatch. They do not have an influence on the arm movements during gait. The arm can thus move naturally, which is in strong contrast to the hand-case covered in this work. Due to the current rise of smartwatches, a combination with the sensoric input of these devices could prove helpful for the detection of ascend and descent on stairs. Since these smartwatches are often additionally equipped with a heart rate monitor, this combination could be extended to include the work done by [64], which uses these vitals as input for activity recognition.

The technique based on statistical features achieved a good accuracy on the pocket-case, and a poor accuracy on the hand-case. The benchmarks of both MFCC and AR-Coefficients showed that these features in isolation are performing better than when the whole set of features is combined. This suggests that some of the included features confuse the classifier instead of contributing to an improved separation. Employing a feature selection approach, as some other works with similar approaches have done [33,65], could prove helpful to find a better set of features for an efficient separation. This would additionally also reduce computational complexity, since fewer features would have to be computed in the first place.

For wider practical uses, the activity recognition should finally also support activities such as using elevators and escalators. These activities would greatly benefit from barometers. However, research has also been done on the detection of elevators by making use of the magnetometer sensor [66]. This could allow supporting these activities, while still only relying on commonly available sensors.

## Figures and Tables

**Table 1:** Overview of the metrics calculated as virtual sensors for the approach based on analytical transformations. For each metric, the table lists whether it is calculated on accelerometer, gyroscope, using a window, if it is calculated per sensor channel, as well as the resulting dimensions.

Virtual Sensor (Metric)	For Accelerometer	For Gyroscope	Calculated per Sample	Calculated per Channel	Channels (Dimensions)
Magnitude	✓	-	✓	-	1
Standard Deviation	✓	✓	-	✓	3
Root mean square	✓	✓	✓	✓	3
Inclination	✓	-	✓	-	2

**Table 2:** Overview of the features in use for the statistical feature approach, with an assignment to the sensors on which they are calculated. Dimensions separated by a slash represent the number of resulting dimensions for raw accelerometer data and data in the earth coordinate system on the left side, and accelerometer data in the two-channel coordinate system on the right side of the slash.

Features	Calculated per Channel	Calculated on Sensor	Channels (Dimensions)
MFCC	yes	Accelerometer	$\left(3/2\right)\;\cdot \;n_{\mathrm{mel}}$
		Gyroscope	$3\;\cdot \;n_{\mathrm{mel}}$
		Accelerometer Magnitude	$n_{\mathrm{mel}}$
		Gyroscope Magnitude	$n_{\mathrm{mel}}$
AR-Coefficients	yes	Accelerometer	(3/2)·|q|
SMA	no	Accelerometer	1
		Gyro	1
Integration	yes	Gyroscope	3
Variance	yes	Inclination	2
		Acceleration Magnitude	1
		Gyroscope Magnitude	1
Max	yes	Inclination	2
		Inclination Gradient	2
Min	yes	Inclination	2
		Inclination Gradient	2
Entropy	yes	Acceleration	3/2
		Gyroscope	3
Correlation	no	Acceleration	3/1
		Gyroscope	3
75th Percentile	yes	Acceleration Magnitude	1
		Gyroscope Magnitude	1

**Table 3:** Overview of all final benchmark results with average classification accuracy and average F1-score (in brackets) for the chosen configuration of each approach. The last column shows the configuration’s hyperparameters, as well as the γ trained for outlier detection.

Approach	KNN		SVM		Outlier Detect	Configuration
	Pocket	Hand	Pocket	Hand		
Analytical Transform	92.4% 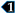 (91.6%)	76.4% 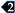 (89.1%)	96.0% 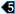 (95.2%)	84.1% 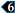 (85.3%)	95.5% (γ=0.005)	PCA 5-dim, $w_{\mathrm{seq}}=2s$, raw acceleration
Codebook	95.5% 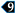 (98.1%)	68.1% 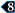 (88.3%)	98.4% 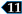 (99.1%)	76.6% 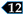 (91.5%)	86.0% (γ=15.0)	σ=0.25, |C|=32, $w_{\mathrm{sub}}=32,w_{\mathrm{seq}}\approx 2s$, raw acceleration
Statistical Features	95.5% 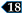 (98.3%)	72.2% 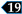 (87.5%)	95.4% 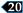 (98.3%)	69.9% 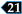 (87.6%)	92.4% (γ=4.55)	LDA 5-dim, $w_{\mathrm{seq}}=2.5s$, $n_{\mathrm{mel}}=10$, ψ=1.6, |q|=5, two-channel acceleration
